# Analysis of Fox genes in *Schmidtea mediterranea* reveals new families and a conserved role of *Smed-foxO* in controlling cell death

**DOI:** 10.1038/s41598-020-80627-0

**Published:** 2021-02-03

**Authors:** Eudald Pascual-Carreras, Carlos Herrera-Úbeda, Maria Rosselló, Pablo Coronel-Córdoba, Jordi Garcia-Fernàndez, Emili Saló, Teresa Adell

**Affiliations:** 1 Department of Genetics, Microbiology and Statistics and Institute of Biomedicine, Universitat de Barcelona, Barcelona, Catalunya Spain; 2 Institut de Biomedicina de la Universitat de Barcelona (IBUB), Universitat de Barcelona, Barcelona, Catalunya Spain

**Keywords:** Developmental biology, Evolution, Genetics, Stem cells

## Abstract

The forkhead box (Fox) genes encode transcription factors that control several key aspects of development. Present in the ancestor of all eukaryotes, Fox genes underwent several duplications followed by loss and diversification events that gave rise to the current 25 families. However, few Fox members have been identified from the Lophotrochozoa clade, and specifically from planarians, which are a unique model for understanding development, due to the striking plasticity of the adult. The aim of this study was to identify and perform evolutionary and functional studies of the Fox genes of lophotrochozoan species and, specifically, of the planarian *Schmidtea mediterranea*. Generating a pipeline for identifying Forkhead domains and using phylogenetics allowed us the phylogenetic reconstruction of Fox genes. We corrected the annotation for misannotated genes and uncovered a new family, the QD, present in all metazoans. According to the new phylogeny, the 27 Fox genes found in *Schmidtea mediterranea* were classified into 12 families. In Platyhelminthes, family losses were accompanied by extensive gene diversification and the appearance of specific families, the A(P) and N(P). Among the newly identified planarian Fox genes, we found a single copy of *foxO,* which shows an evolutionary conserved role in controlling cell death.

## Introduction

Forkhead box (Fox) genes belong to the ‘winged helix’ superfamily of transcription factors (TF) with a specific DNA-binding domain referred to as the Forkhead domain (FKH), with approximately 100 Aa. In Metazoa, Fox genes are expressed in a specific spaciotemporal manner during development, and control essential processes as cell death, cell cycle and stem cell differentiation into specific cell lineages and populations^1–3^. Thus, Fox genes play a major role during embryonic and postembryonic development, including: gastrulation, lifespan, immune system regulation or tissue differentiation and maintenance^1^. In humans, the lack of some Fox genes leads to embryonic lethality or developmental diseases such as Parkinson’s, defects in the immune system, speech and language learning or cancer^1,2,4,5^.

Currently, over 2000 Fox proteins have been identified in 108 species of fungi and metazoans, including a vast number of Phyla, such as Choanoflagellata^6^, Ctenophora^7^, Placozoa^8^, Porifera^9^, Cnidaria^10^, Echinodermata^11^, Hemichordata^12^, Cephalochordata^13,14^ and Chordata^14,15^. However, few Fox genes have been identified in lophotrochozoan species, and most functional studies have been only performed in few model organisms such as mice^16–18^, *Drosophila melanogaster*^19,20^ or *C. elegans*^21^*.* Planarians are Lophotrochozoans well known for their astounding ability to regenerate any body part and change their size according to food availability. Such tissue plasticity is due to the presence of adult stem cells (neoblasts) that can give rise to all differentiated cell types, which is accompanied by the continuous activation of the intercellular signalling mechanisms^22–24^. Planarians’ phylogenetic position and plasticity makes them an interesting model for investigating the Fox family at an evolutionary and functional level.

Fox genes are currently phylogenetically classified and grouped into 25 families (A to S)^2,25^. The different gains and losses of Fox families have shaped the history of Fox family evolution, such as the division of family Q into Q1 and Q2, N into N1/4 and N2/3, L into L1 and L2 or J into J1 and J2/3^14^. Another example of gain is the S family, which seems to evolve by duplication of the C family and is specifically found in vertebrates^11,26^. Fox family losses have also been reported, such as the AB in vertebrates^15^ or E, H, I, J2/3, M and Q1 in Ecdysozoa^14^.

The aim of this study was to identify and classify the Fox genes of the planarian species *Schmidtea mediterranea* (*Smed*), along with the Fox genes of other lophotrochozoan species whose genome or transcriptome is currently available^27–32^. Previous studies of Fox genes expression and function in *Smed* showed that they were tissue specific and participated in its maintenance. The essential role of *foxA* in the maintenance of the pharynx and endodermal tissue has already been identified in planarians^33^, similar to its role in early endoderm development in vertebrates^34–36^, or the role of J1 paralogs in ciliogenesis^37^, similar to the one described in vertebrate species as mice, chickens or frogs^38,39^. However, other important Fox families such as the O family, related with metabolism, growth and aging^40,41^ had not been identified in planarias.

Through generating a pipeline for identifying Forkhead domains we identified and annotated 27 Fox genes in *Smed,* 18 of which are firstly reported here. Phylogenetic analyses allowed us to classify *Smed* Fox in 12 families. The integration of the newly identified Fox from *Smed* and other lophotrochozoans and Platyhelminthes with all reported Fox genes allowed for the identification of the QD family, a new family which appears to originate after the split of sponges from the rest of the eumetazoans. Most of the Fox families also originated during this period, which was followed by various loss events and some diversification. Specifically, Platyhelminthes suffered a huge gene family loss followed by gene diversification originating specific families: A(P) and N(P). Finally, we identified a single copy of the *foxO* gene in planarians and demonstrated its conserved role in controlling cell death.

## Results

### *Schmidtea mediterranea* presents 27 Fox genes that can be classified in 12 families

**Figure 1:**
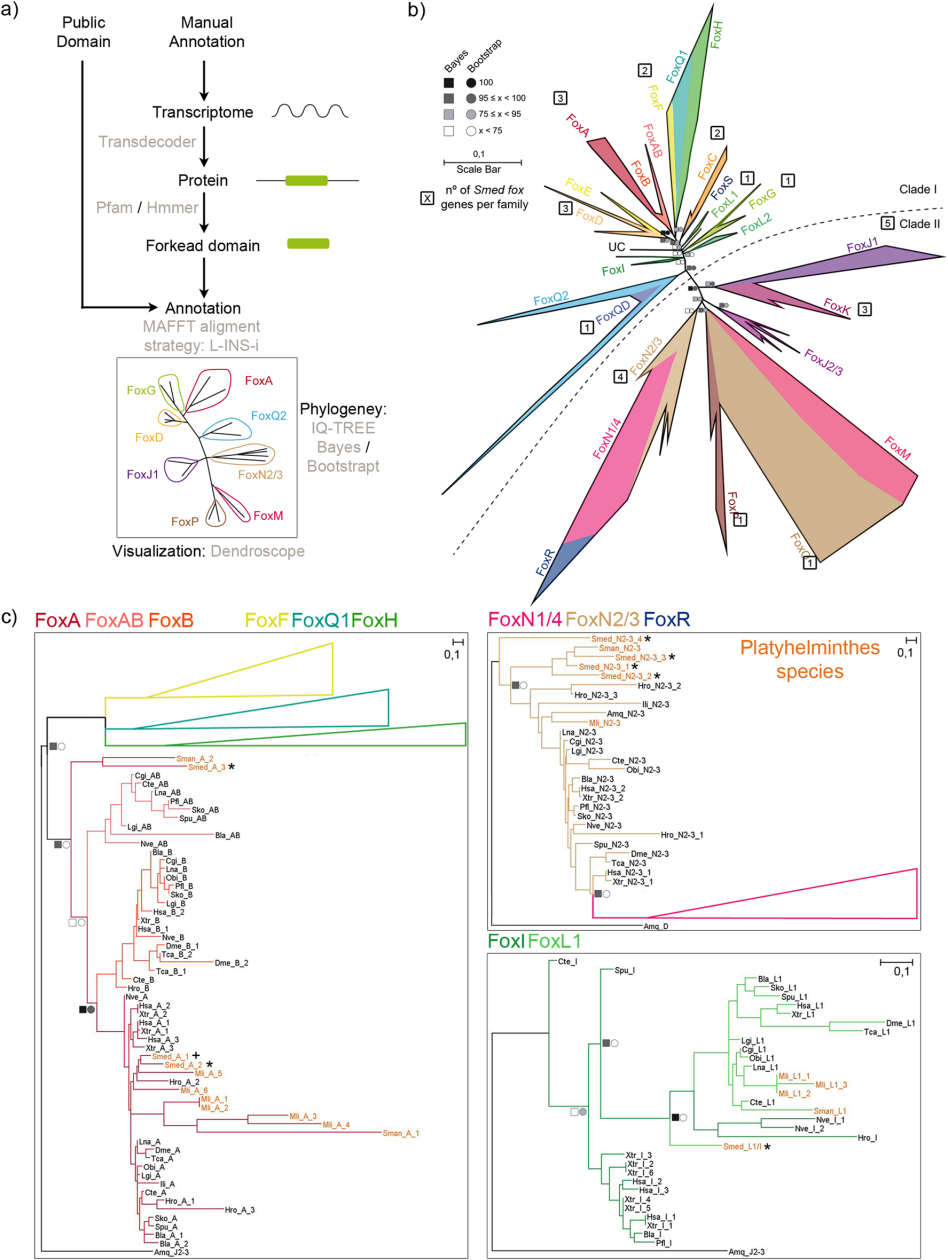
Fox family evolution in Metazoa reveals 27 Fox genes in *Schmidtea mediterranea* divided in 12 families. (**a**) pipeline annotate Fox genes. (**b**) The ML phylogenetic trees based on FKH. Number of genes per family in *Schmidtea mediterranea* is indicated inside a square next to each family. At nodes, values for the approximate Bayes (square) and Likelihood (circle) ratio test are shown. Colour indicates % of confidence. Family tree branches were collapsed at the base of the common node. One gene was unclassified in any family (UC). Dashed line divides Clade I and Clade II Fox genes. (**c**) For each node-sharing families, a phylogenetic tree was created using an *Amq* gene from the opposite clade as out group. Family branches are painted with the same colour as they are represented in the trees. Platyhelminthes genes are coloured light orange. Dark crosses indicate previous characterized genes and dark asterisks indicate new fox characterized in *Schmidtea mediterranea* (*Smed*). Aminoacidic sequences used are found in S1 File. Scale indicates expected aminoacidic substitution per site.

With the aim to identify all Fox genes of *Smed*, we developed a pipeline for identifying Forkhead domains (FKH) using the available FKH from Pfam in combination with TransDecoder and HMMER (Fig. 1a, see “Methods”). As a result, we found 27 distinct genes that contained this domain in the planarian genome^27^. To determine which family each of these genes belonged to, we performed a phylogenetic analysis using the FKH domain of the Fox genes of an additional 20 species across metazoans, including several lophotrochozoans, to better resolve the *Smed* Fox groups (Fig. 1a and “Methods”). The analysis resulted in the classification of the 27 FKH-containing *Smed* genes into 12 Fox families (Figs1b,c, S1, S2, S3). The complete information and new annotation regarding each FKH-containing gene identified is provided in the supplementary materials (S1 File), along with the raw tree (S2 File).

The phylogenetic tree (Fig. 1b) shows that 5 out of these 12 families belong to Clade II, which is argued to be the ancestor clade^42,43^; and 7 belong to Clade I. To better visualise the different Fox genes in each cluster, we inferred a series of new phylogenetic trees including only the genes from closely related families (Figs. 1c, S2, S3). Using this visualisation, we found some *Smed* Fox genes that were not properly classified: a *Smed* Fox gene that clusters between the L1 and the I families (*Smed-foxL1/I*) (Fig. 1c), a *Smed* Fox gene clustering as a sister group of the A, AB and B families (*Smed-foxA3*) (Figs. 1c, S2), and a *Smed* Fox clustering as a sister group of the N2/3 family (*Smed-foxN2/3–4*) (Fig. 1c, S3). Furthermore, we can observe how the Q2 family, widely described in many species^12,44–46^ has a branch populated with several genes that cluster with a divergent Q2 gene known as *foxQD* in *Saccoglossus kowalevskii* (*Sko*)^12^. We consider this branch to be a new family (Fig. S2) which we called QD, due to the FKH-containing gene originally described in *Sko*. A *Smed* Fox gene belongs to this family (*Smed-foxQD*).

**Figure 2:**
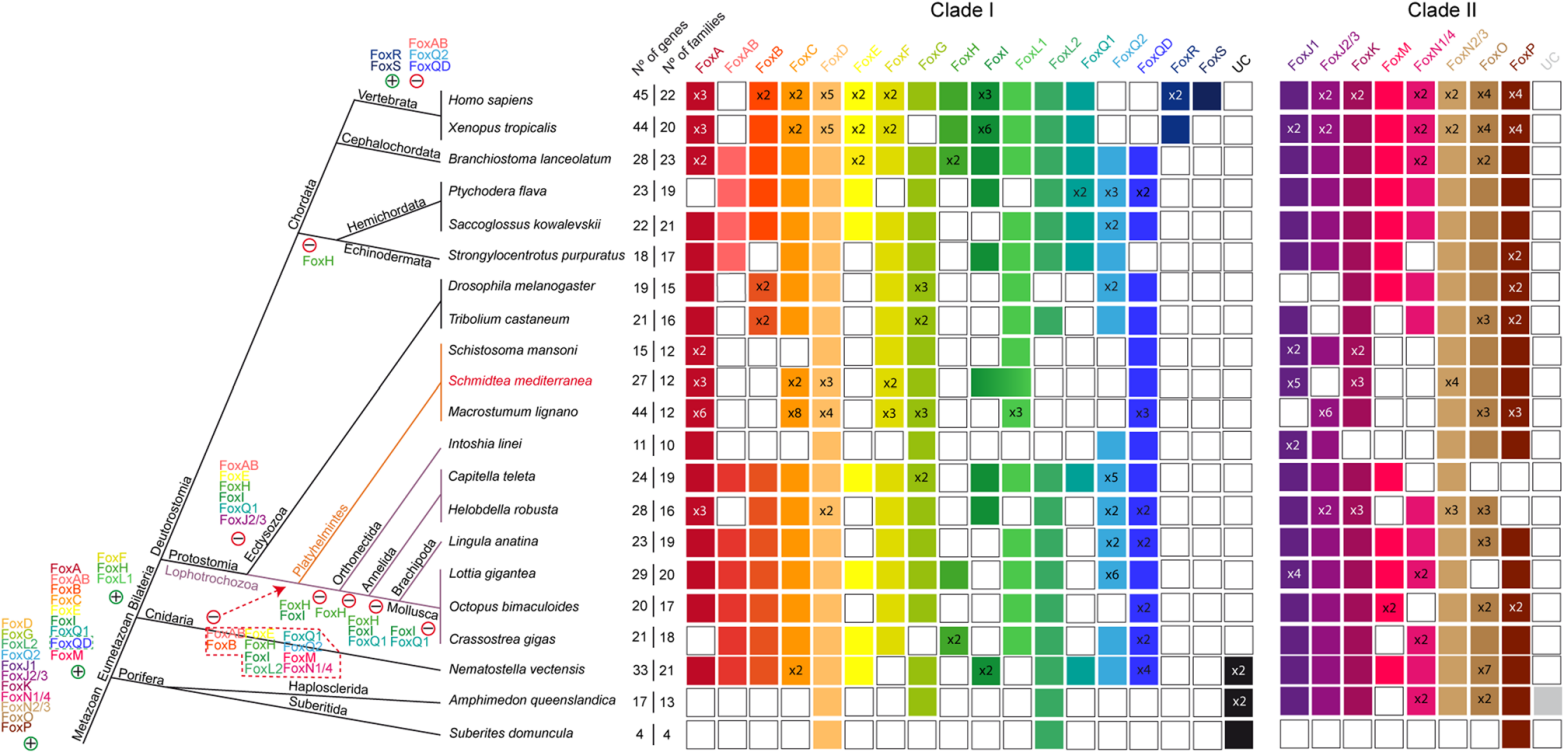
Distribution of Fox homologs in Metazoan clade indicating gene and family losses and some gene duplications in *Schmidtea mediterranea*. Coloured boxes indicate the presence of an ortholog based on the phylogenetic analysis. When there were no evidences of ortholog, the box remained white. A number (x Nº) inside a box indicates paralogs per family and species. Families are divided in Clade I and II. Number of genes and number of families per species are indicated. Metazoan^47^ and Lophotrochozoa^48^ phylogenies were used. Light purple lines indicate lophotrochozoan species and within light orange indicate Platyhelminthes superphylum species. Gains (+) and losses (−) of genes are placed next to each clade. Main Clade I Fox acquisition was at the base of Eumetazoa and different events of gains and losses happened through evolution. Specifically, many families were lost in Platyhelminthes (red dashed polygon).

Focusing on the presence of Fox genes in each family, we can observe that despite the number of Fox genes in *Smed* has remained similar to the rest of lophotrochozoans (see purple lines in Fig. 2), the number of families with representatives of *Smed* and the other two Platyhelminthes (*Schistosoma mansoni* and *Macrostumum lignano*) has decreased. Particularly, Platyhelminthes seem to have lost the AB, B, E, H, I, Q1, Q2, M and N1/4 families (red dashed square in Fig. 2). This suggests a huge family loss at the base of Platyhelminthes phylum (orange lines in Fig. 2) coupled with an expansion of the Fox number in specific families.

### Platyhelminthes present specific Fox subfamilies: FoxA(P) and FoxN(P)

**Figure 3:**
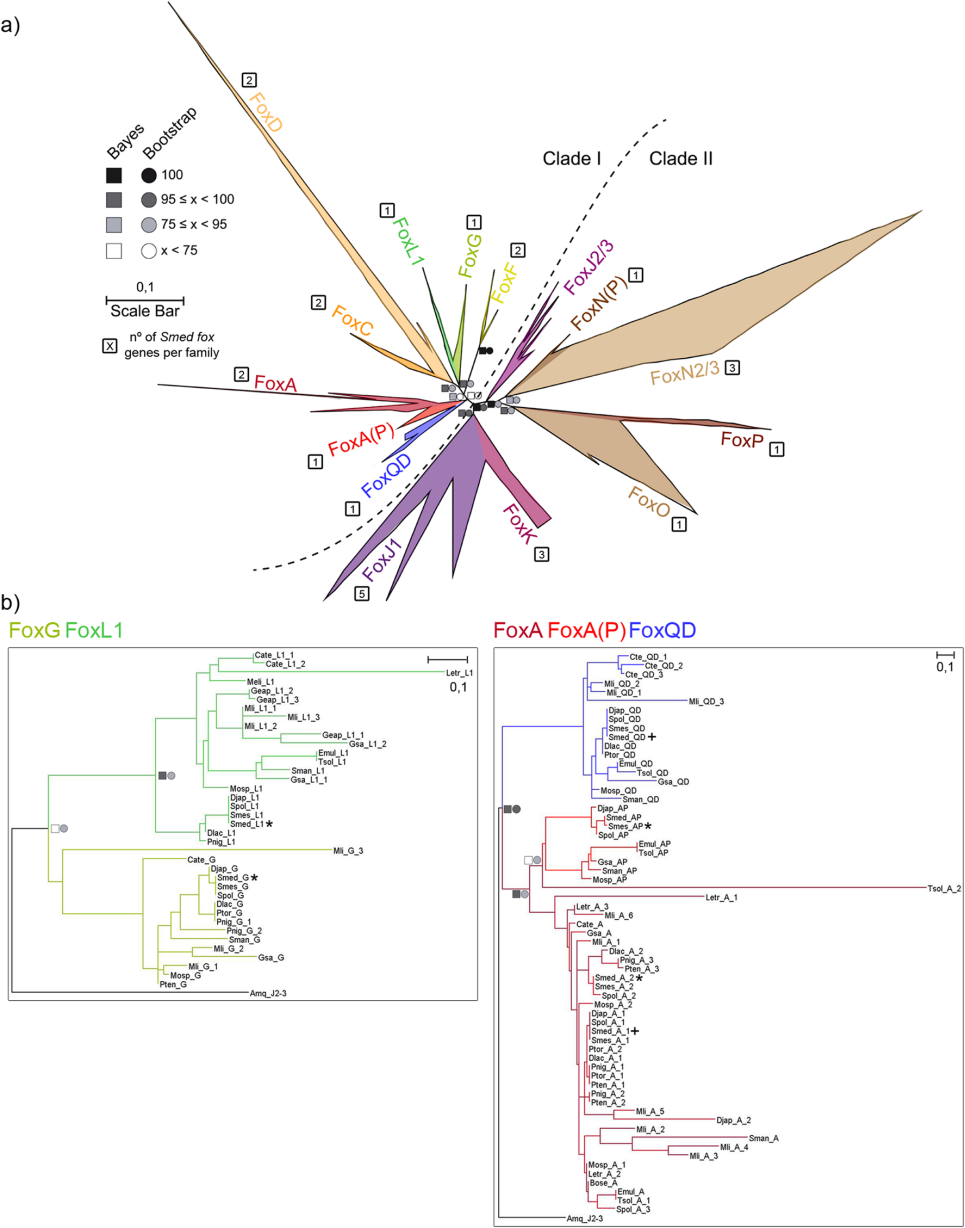
Fox family evolution in Platyhelminthes indicates family diversification. (**a**) The ML phylogenetic trees based on FKH of Fox family evolution in Lophotrochozoan clade. Number of genes per family in *Schmidtea mediterranea* is indicated inside a square next to each family. At nodes, values for the approximate Bayes (square) and Likelihood (circle) ratio test are shown. Colour indicates % of confidence. Family tree branches were collapsed at the base of the common node. Dashed line divides Clade I and Clade II Fox genes. (**b**) For each node-sharing families, a phylogenetic tree was created using an *Amq* gene from the opposite clade as out group. Family branches are painted with the same colour as they are represented in the trees. Dark cross indicates previous characterized gene and dark asterisk indicates new fox characterized in *Schmidtea mediterranea* (*Smed*). Aminoacidic sequences used are placed in S3 File. Scale indicates expected aminoacidic substitution per site.

To further investigate the phylogeny of the unclassified *Smed* Fox genes (*Smed-foxL1/I*, *Smed-foxA3* and *Smed-foxN2/3–4*) we performed a second phylogenetic analysis focused on Platyhelminthes data. Repeating the same pipeline previously described, we identified the FKH-containing genes from a total of 19 Platyhelminthes species (including *Smed*), 8 of which belong to the Tricladida order, to which *Smed* belongs (Fig. 3a). Platyhelminthes Fox data can be found in the S3 File and the raw tree can be found in the S4 File. As previously, we also performed additional phylogenetic trees of close-related families to better visualize each family (Figs. S1, S3b, S4, S5). This analysis allowed us to properly classify the FoxL1/I gene into the L1 family (Fig. 3b), which seems to be slightly divergent in the Tricladida Order, and thus we renamed it as *Smed-foxL1.* Furthermore, the new analysis allowed the identification of two new subfamilies only present in Platyhelminthes to which the *Smed-foxA3* and the *Smed-foxN2/3–4* genes belonged. Thus, we renamed them as *Smed-foxA(P)* (Fig. 3b) and *Smed-foxN(P)*, respectively (P meaning specific of Platyhelminthes) (Fig. S5). In this analysis the N subfamily was found to be specific to Triclads, while the A(P) subfamily was also found in other Platyhelminth orders.

Based on these analyses we have identified and classified all FKH containing genes of *Smed*, including the ones already published, which in some cases have been reclassified according to our analysis. Thus, the previous *Smed-foxQ2*^49^ is now classified as *Smed-foxQD*, and the previous *Smed-Albino*^50^ gene is now renamed as *Smed-foxP*. The new classification of all *Smed* Fox genes can be found in Table 1 (also in S1 Table). We did not to relate the subclassification of family genes between species, since it could cause a misleading annotation (i.e. *foxD2* genes of *Smed* and *Hsap* are not directly homologs). The analysis of their protein domains shows that in addition to the FKH domain, the K family genes also contained the Forkhead associated domain (FHA) and the P family also showed a related coiled coil. Most of the proteins were enriched in nuclear localization signal (NLS) and/or nuclear export signal (NES), in accordance with their function as TFs (Fig. S6).

**Table 1:** Fox genes in *Schmidtea mediterranea*.

Family	New gene name	Previous name	Gene Bank ID
A	*Smed-foxA1*	*Smed-FoxA*	JX010556 / BK013013
	*Smed-foxA2*		BK013028
	*Smed-foxA(P)*		BK013026
C	*Smed-foxC1*		BK013019
	*Smed-foxC2*		AY967492 / BK013022
D	*Smed-foxD1*	*Smed-FoxD*	KC577557 / BK013024
	*Smed-foxD2*		BK013027
	*Smed-foxD3*		BK013021
F	*Smed-foxF1*	*Smed-FoxF*	KJ934801 / BK013018
	*Smed-foxF2*	*Smed-FoxF-1*	BK013034
G	*Smed-foxG*		BK013020
L1	*Smed-foxL1*		BK013023
Q/D	*Smed-foxQ/D*	*Smed-FoxQ2*	JX091076 / BK013031
J1	*Smed-foxJ1-1*	*Smed-FoxJ1-1*	JX842843
	*Smed-foxJ1-2*	*Smed-FoxJ1-2*	JX842844
	*Smed-foxJ1-3*	*Smed-FoxJ1-3*	JX842845
	*Smed-foxJ1-4*	*Smed-FoxJ1-4*	JX842846
	*Smed-foxJ1-5*		BK013017
K	*Smed-foxK1*		BK013030
	*Smed-foxK2*		BK013032
	*Smed-foxK3*		BK013035
N2/3	*Smed-foxN2/3–1*		BK013014
	*Smed-foxN2/3–2*		BK013016
	*Smed-foxN2/3–3*		BK013029
	*Smed-foxN(P)*		BK013015
O	*Smed-foxO*		BK013025
P	*Smed-foxP*	*Smed-Albino*	KX531078 / BK013033

New and previous names of Fox genes in *Smed* are shown, with their corresponding GeneBank Ids.

**Figure 4:**
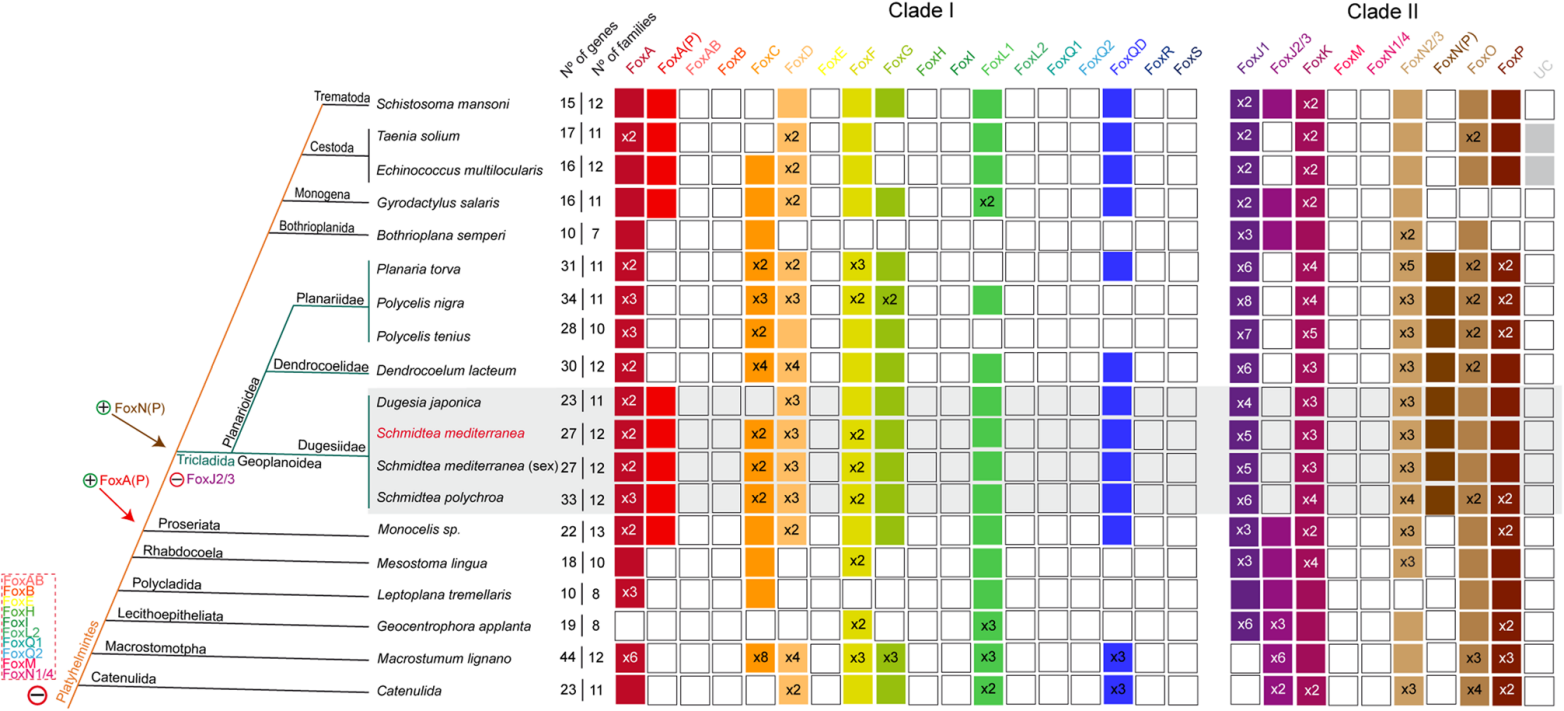
Distribution of Fox homologs reveals huge family loss and gene diversification in Platyhelminthes. Coloured boxes indicate the presence of an ortholog based on the phylogenetic analysis. When there were no evidences of ortholog, the box remained white. A number (x Nº) inside a box indicates paralogs per family and species. Families are divided in Clade I and II. Number of genes and number of families per species are indicated. Species were classified into Platyhelminthes and Tricladida phylogeny accordingly to^28^ and^51^, respectively. Light orange indicates Platyhelminthes species within turquoise lines indicate Tricladida species; grey box indicates Dugesiidae family. Species belonging to the Tricladida order show different losses, gains and specialization events. Gains (+) and losses (−) of genes are indicated. Specifically, many families were lost in Platyhelminthes (red dashed polygon). FoxA(P) and FoxN(P) origin seems to predate Proseriata and Tricladida order, respectively.

Thanks to this new analysis, we could also confirm the loss of several Fox families in planarians (the AB, B, E, H, I, Q1, Q2, M and N1/4 families) and we could determine that most of this family losses found in planarians predate the emergence of the Platyhelminthes phylum. Besides the family losses earlier mentioned (Fig. 2), Tricladida additionally lost the J2/3 family. Interestingly, Tricladida (see turquoise lines in Fig. 4) have doubled the FKH-containing genes compared to the other Platyhelminthes, while the number of families remained constant, supporting an intrafamily diversification of Fox genes in this group (Fig. 4).

**Figure 5:**
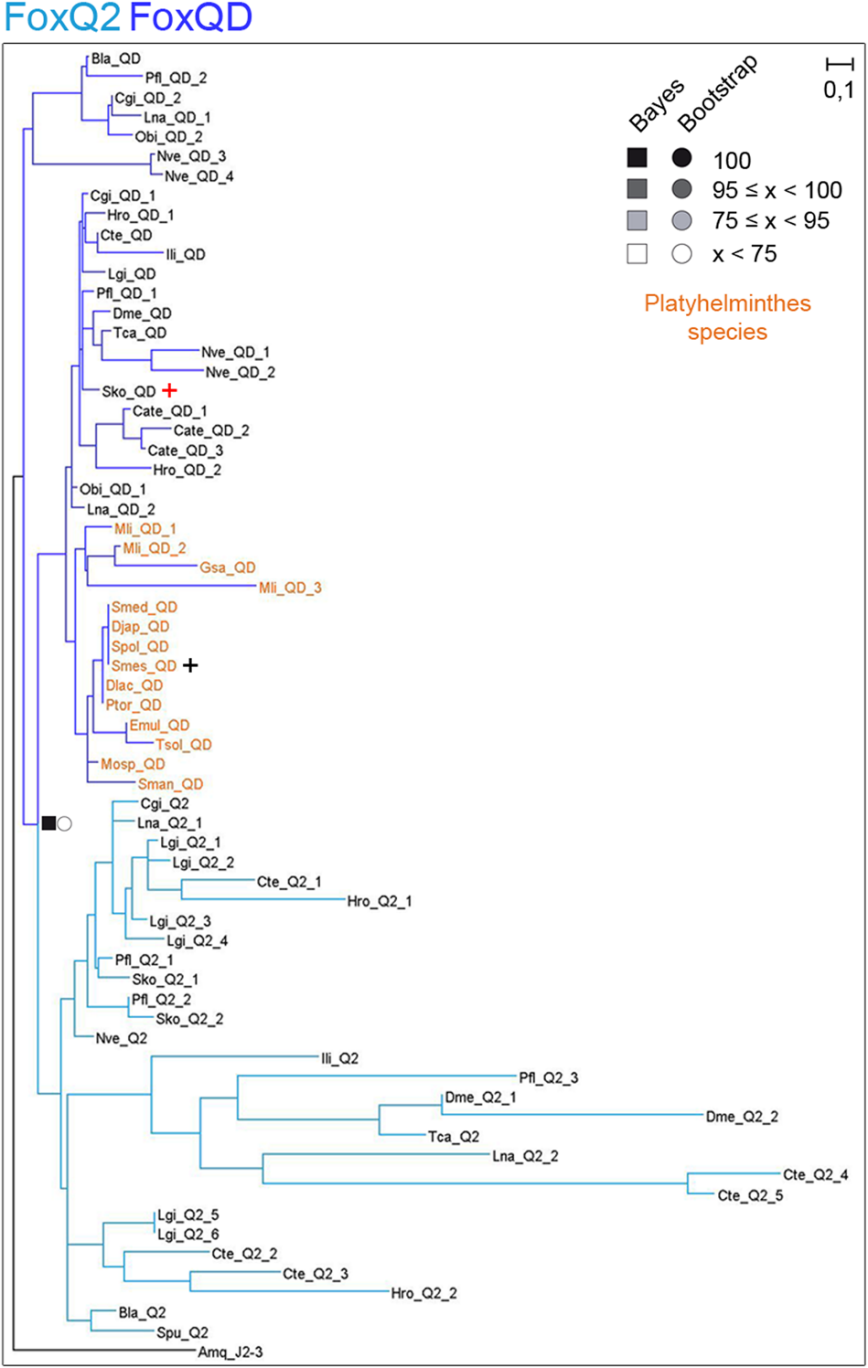
New family FoxQD is broadly found in Metazoa but missing in Vertebrata and Porifera. The ML phylogenetic trees based on FKH of Q2 and QD family evolution in all metazoan species studied in this work. At nodes, values for the approximate Bayes (square) and Likelihood (circle) ratio test are shown. Colour indicates % of confidence. An *Amq-J2/3* from the opposite clade was used as out group. Family branches are painted with the same colour as they are represented in the trees. Dark asterisk indicates a new fox characterized in *Schmidtea mediterranea* (*Smed*). Red cross indicates *Saccoglossus kowalewski foxQD* gene. Platyhelminthes QD genes are coloured light orange. Aminoacidic sequences used are placed in S1 and S3 File. Scale indicates expected aminoacidic substitution per site.

To note, the new QD family found in this study was found to be present in all Platyhelminthes, while the Q2 family is lost in all of them. To better decipher the relation between the new QD family and Q2 family, we performed a new phylogenetic analysis (Fig. 5). Increasing the number of species used outputted more confident Bayes and Bootstrap node values, supporting that a new Fox family has been uncovered, which is present in most Metazoa, including Platyhelminthes. Some genes misannotated in other families, such as Q2, B and I were reannotated as QD (Table 2, S1 File).

**Table 2:** New annotation of QD genes in different metazoan species.

Species	nº FoxQD	Annotation		
		New	Re	Previous
*Branchiostoma lanceolatum*	1		x	FoxD3
*Ptychodera flava*	2	x		
*Saccoglossus kowalevskii*	1			
*Drosophil melanogaster*	1		x	Non annotated
*Tribolium castaneum*	1		x	FoxA1_B-like
*Schistosoma mansoni*	1	x		
*Schmidtea mediterranea*	1		x	FoxQ2
*Macrostumum lignano*	3	x		
*Intoshia linei*	1	x		
*Capitela teleta*	1		x	FoxQ2_Lb
*Helobdella robusta*	2	x		
*Lingula anatina*	2	x		
*Lottia gigantea*	1		x	FoxQ2b
*Octopus bimaculoides*	2	x		
*Crassostrea gigas*	2		x	FoxB/FoxI
*Nematostella vectensis*	4	x		

Number of paralogs per species is indicated. They are classified as New or Re-annotated genes. In this case, the previous family is shown.

### *Schmidtea mediterranea* Fox genes appear not to be linked in the genome and to have drifted through evolution

**Figure 6:**
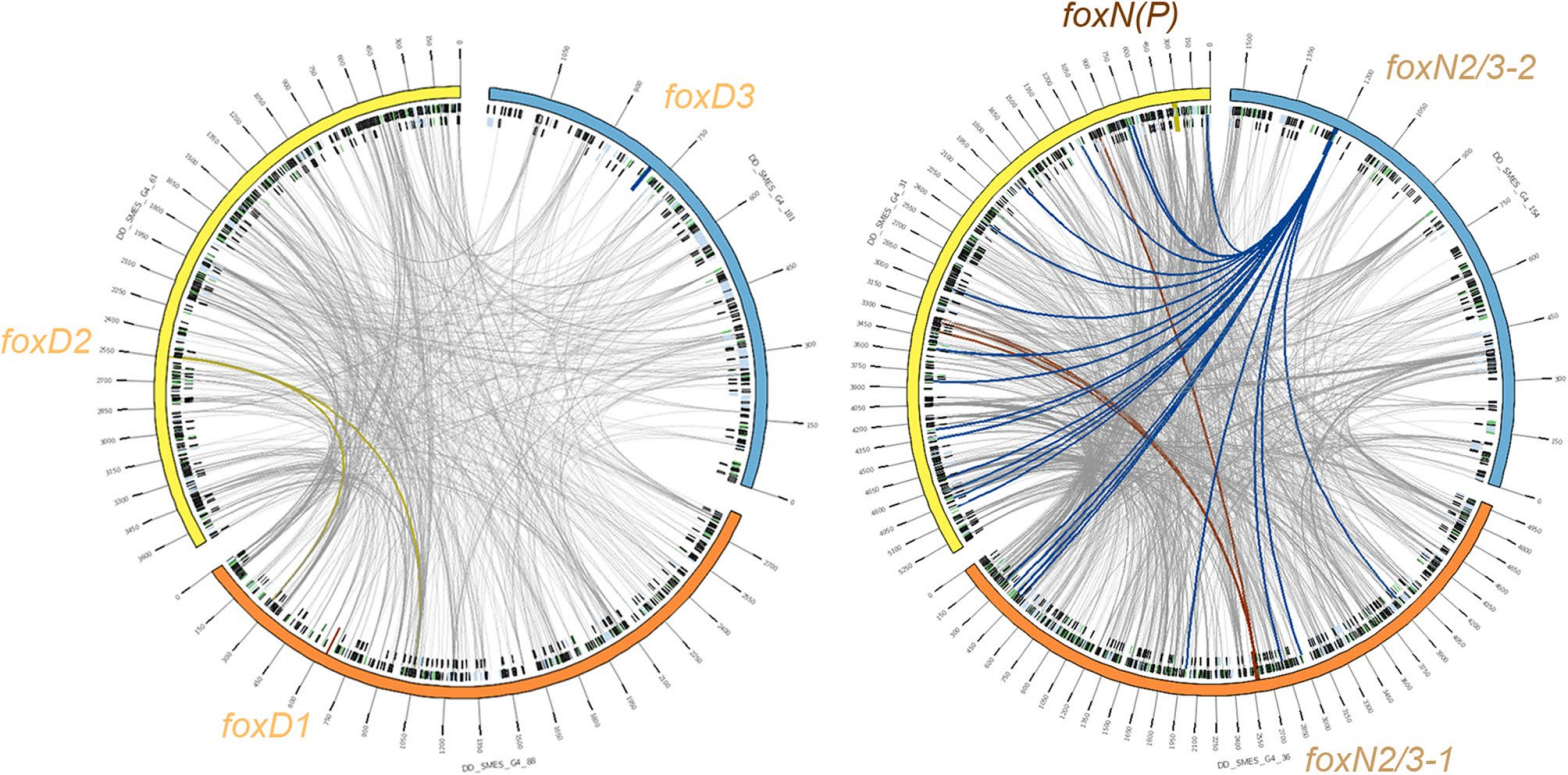
Fox paralogs do not present syntenic relationships in *Smed* genome. Alignments between scaffolds containing same-family Fox genes are represented with Circos. The Circos representation is composed of two tracks: In the outer ring the scaffolds containing Fox genes are labelled with their name (each tick representing 150 kb); in the inner ring, the repeating elements^29^ coloured in green (LINEs), blue (TLR) and black (simple repeats and other). Repeats are filtered to be shown only when greater than 1 kb. Grey lines connecting the scaffolds are the representation of the alignments, filtered to be shown only when greater than 1 Kb. In each scaffold, the region corresponding to the Fox gene (+ -5 Kb) is represented as a perpendicular darker region, and all the links that fall onto it are coloured accordingly.

Regarding the relative position of Fox genes in the genomes of Metazoa, we can see how some of them typically present a linkage such as in the case of the families D-E or C-F-L1-Q1, which in other species is shown to cover different interval regions, from 20 to 300 kb^26,52, 53^. When comparing their genomic position relative to other coding genes, Irimia et al.^54^ demonstrated that some of the Fox genes had retained microsynteny across metazoans with a variety of genes. Although the *Smed* genome^27^ is not assembled at chromosome level, we examined the genomic neighbourhood of *Smed* Fox genes. The only genes present in the same scaffold were *foxD2* and *foxA1* with ~ 187 kb of distance between them (S1 Table). Although the distance is less than the 0.1% of genome size^52^ there are no other reports of A-D family linkage, meaning that no canonical Fox genes linkages are found in this assembly version of *Smed* genome. In order to verify if, despite this, there was some level of microsynteny conservation, we took an orthology-based approach similar to the one used to identify orthologous lincRNAs between distant species^55^ using humans as a comparison, as well as manually checking the existence of already described microsyntenic pairs. In both cases, we found no conserved microsynteny (S5 File). Additionally, we decided to perform whole-genome alignments between the different scaffolds to examine the inter-paralog syntenic relationships (Figs. 6, S7). However, the synteny seems to be broken in all cases with most of the alignments falling exclusively into repeating elements such as LINEs.

Our data suggest that the Fox families found to be linked in other species (C-F-L1-Q1 and D-E)^26,52^ are not linked in *Smed*, and *Smed* Fox genes show no microsynteny with described genes. However, this analysis should be repeated when the genome of *Smed* is completely assembled.

### *Smed-foxO* controls cell death in planarians

The expression pattern and function of most *Smed* Fox genes remain unstudied. We performed an exhaustive analysis of their expression pattern by in situ hybridization and by an *in-silico* search in the SCSeq databases^56,57^, as well as a functional screening through RNAi. The results show a tissue-specific pattern of expression for most of the genes which could give clues about their function (Figs. S8–S10, S1 Table). RNAi inhibition produced regenerative defects that were mild in some of them (Fig. S11) but obvious in others, which could not regenerate proper eyes nor CNS (Fig. S12). For instance, *foxD3* was expressed in GABAergic neurons according to the SCSeq databases (Fig. S8) and when inhibited produced strong regenerative defects in the brain (Fig. S12, S1 Table). Both Platyhelminthes/planarian specific *foxA(P)* and *foxN(P)* showed strong phenotype after inhibition (Fig. S12). *foxA(P)* was expressed in ‘activated early epidermal progenitors’ according to the SCSeq databases (Fig. S8) and when inhibited animals could hardly regenerate nor anterior or posterior wounds, giving rise to small animals (Fig. S12). *foxN(P)* was expressed in brain branches and epidermal cells (Fig. S9), and when silenced animals did not regenerate eyes and presented a smaller brain, suggesting that RNAi animals presented neural regenerative defects (Fig. S12).

**Figure 7:**
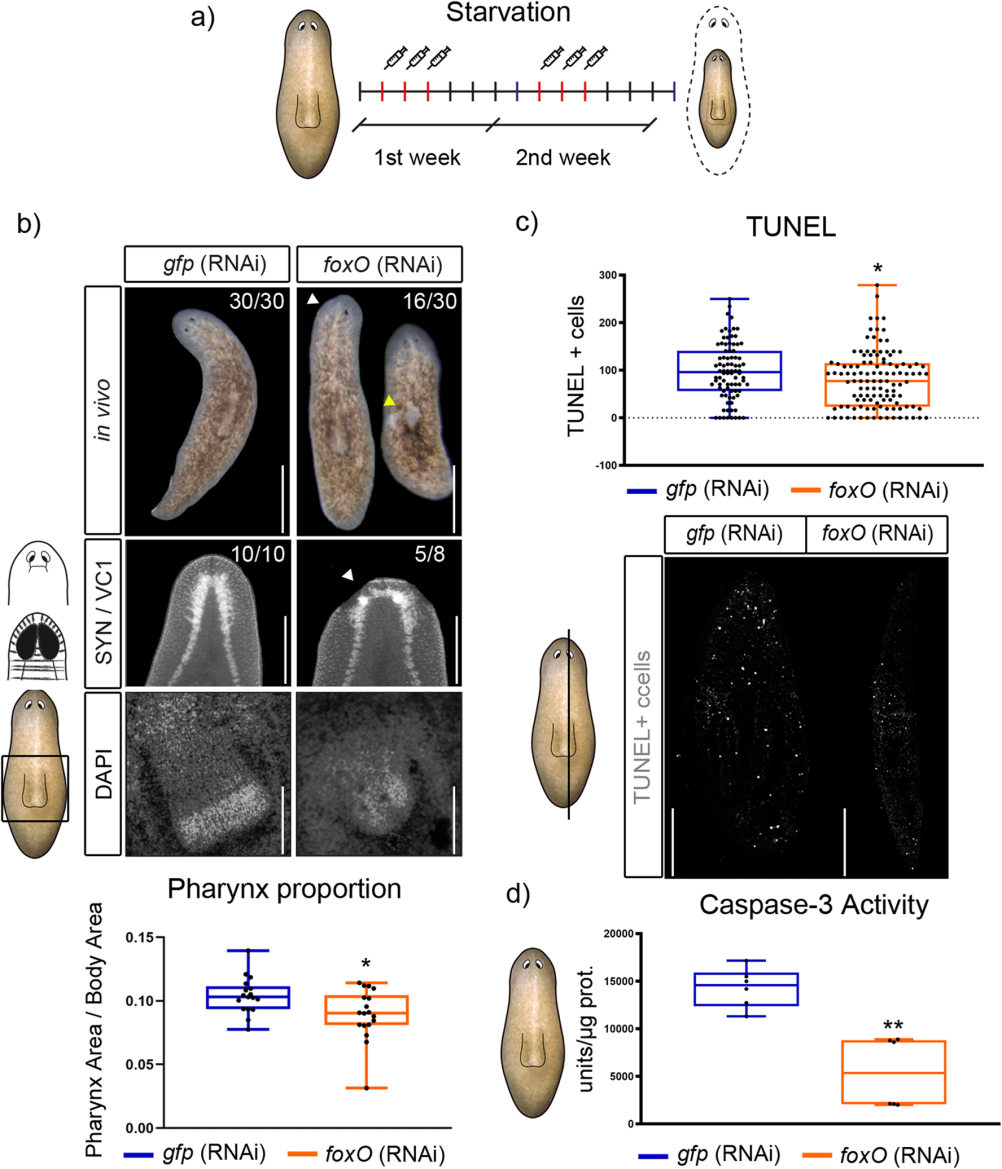
Starving *foxO* (RNAi) animals show tissue disruption and a reduction of cell death. (**a**) Schematic depicting RNAi procedure. (**b**) 50% of in vivo* foxO* knockdown animals presented unpigmented regions (white arrow), neural tissue disappearance (yellow arrow in synapsin immunoassayed animals, and pharynx size reduction, as shown by DAPI staining and relative size quantification (controls, n = 17; RNAi, n = 18; *P < 0.05). (**c**) Quantification of TUNEL + cells on transversal sections show a reduction of positive cells in *foxO* (RNAi) animals compared to controls (controls, n = 86; RNAi, n = 117; *P < 0.05). (**d**) Quantification of caspase-3 activity in *foxO* (RNAi) animals and controls shows cell death reduction (controls, n > 6; RNAi, n > 6; **P < 0.01). Scale bars: b up and mid = 100 µm and down = 10 µm; c = 100 µm.

We found particularly interesting the discovery of a unique copy of *foxO,* which is the homolog of *daf-16* in *C. elegans,* known for its role in increasing longevity^58^. Nowadays *foxO* is known to be crucial in controlling metabolism and oxidative stress and also participates in the regulation of genes related to tissue repair and homeostasis^41^. Thus, we wondered whether *Smed-foxO* could have a role in controlling tissue regeneration and homeostasis as well in planarians. First, we found that *Smed* FOXO conserves the three specific sites (Fig. S13a), that under nutrient rich conditions are phosphorylated by AKT and lead to its ubiquitin degradation^59, 60^. To check whether in planarians this mechanism is conserved, we quantified *foxO* levels in fed animals. qPCR quantification demonstrated that *foxO* was down-regulated in fed conditions (Fig. S13b). Altogether, suggested that the AKT-dependent mechanism of *foxO* regulation could be conserved in planarians. Then, we found *Smed-foxO* expressed ubiquitously and according to the SCSeq databases it was present in several cell types, such as neoblasts, neurons, parenchymal, secretory and epidermal cells (Fig. S9–S10, S1 Table). Trough RNAi inhibition, we found that in regenerating animals *foxO* RNAi inhibits the differentiation of the eyes and the proper regeneration of the brain (Fig. S13c).To gain insight into its function in tissue renewal, we inhibited it by RNAi in intact starving animals. qPCR demonstrates *Smed-foxO* mRNA downregulation in *Smed-foxO* (RNAi) animals (Fig. S13d). After two weeks of inhibition half of knockdown animals presented unpigmented zones, areas in which the brown pigment of epidermal or eye cells is lost (Fig. 7a). Analysis of the CNS (anti-synapsin) and the pharynx (DAPI) demonstrates that those structures are smaller and not properly maintained in *Smed-foxO* RNAi animals (Fig. 7b). The problems in tissue turnover could arise by unpaired cell proliferation and/or cell death. To test these possibilities, we analysed M phase cells through PH3 staining, and apoptosis by TUNEL and caspase-3 assay. Cell proliferation was unaffected after two weeks of inhibition (Fig. S13e). However, both TUNEL and caspase-3 assays demonstrate that the apoptotic response that normally takes place in starved planarians, which are shrinking, was inhibited in *Smed-foxO* RNAi animals (Fig. 7c,d). To confirm the pro-apoptotic role of FoxO in planarians we quantified the levels of the pro-apoptotic gene *bak*, which is a reported target of *foxO*^61^. qPCR quantification shows their downregulation in *foxO* (RNAi) animals (Fig. S13f). A decrease in cell death that is not balanced by an increase in cell proliferation must end up with an increase in the number of cells. We found that *foxO* (RNAi) planarians reduced their size equivalently to control animals (Fig. S13g). However, the quantification of cells in the epidermis demonstrates an increase in cell density in the RNAis (Fig. S13h). Furthermore, we observe that epidermal cells are disorganized.

Overall, these results demonstrate that *Smed-foxO* is necessary for planarian cell turnover through the control of cell death but not cell proliferation.

## Discussion

### The Fox genes in Metazoa: a story of early gains and specific losses

The Fox genes evolution has been a field of interest since their discovery. Although quite rich in different families, it seems that the ancestral Fox gene was remarkably similar to the J1 family. This family has been proposed as the original Fox family, giving rise to the rest of families by gene expansion and duplication^42^. Few Fox genes have been identified in choanoflagellates and in sponges^6,52^ and, as seen in Fig. 2, an expansion of the families took place before the origin of cnidarians, when most of the Clade II families and some Clade I families appeared. At the base of bilaterians, new families appeared: F, H and L1 (exclusive of bilaterians); R and S (vertebrate and mammal specific, respectively). This would mean that some of the Fox families are easily lost through evolution, as seems to be the case for H, I, and Q1 families. In agreement with other analysis^43,62^, those families would have appeared early on the metazoans, and then were lost in a species-specific manner several times through evolution (Fig. 2). In addition, our findings support that some clades suffered massive losses, such as Ecdysozoa which has lost seven families, as it has been proposed by Mazes et al.^14^, although the losses in this clade could be less if more organisms where used in the analyses. Nonetheless, the clade with the greatest number of specific losses seems to be Platyhelminthes with ten families lost (Fig. 4). This is in line with other studies that have analysed the evolution of different gene families in Platyhelminthes, such as the Wnt family, which demonstrated a great number of family losses in this clade^63–65^.

### Uncovering the QD family

In this study we identified a new Fox gene family not previously described, the QD, to which some genes misannotated in other families belong to, such as Q2, B and I (Table 2, S1 File). This family originated from a paralog duplication that gave rise to both the already known Q2 family and the new QD family. According to our data, the origin of the QD family could be prior to the eumetazoan radiation although to confirm this, analysis on placozoans and ctenophores would need to be carried out. The QD family is present in almost all taxa studied and is completely lost only in vertebrates (Fig. 2).

Not only the phylogenetic data but also their different expression patterns support that QD and Q2 are different families. While Fox Q2 genes show an evolutionary conserved role in anterior brain development^44,66^, *Sko foxQD* shows a completely different spatiotemporal expression^12^. *Smed foxQD*, which was annotated as *foxQ2*, is not expressed in nor has a role in anterior patterning^49,67,68^. We hope that, much like in the case of the uncovering of the AB family by Yu et al.^13^, this new family can help to elucidate some of the inconsistencies in annotation such as in the case of *Smed* and will also contribute to a better understanding of the function of the different Fox families.

### Planarian-specific Fox: FoxA(P) & FoxN(P)

When analysing the Platyhelminthes tree, we were able to properly classify the outgrouping L1/I *Smed* Fox into the L1 family (Fig. 3b). Furthermore, having more Platyhelminthes in the phylogeny resulted in two families grouping into a new branch (Figs. 3a,b, S4, S5). These were named *foxA(P)* and *foxN(P)* as they appeared to be divergent members of the A and N families. The L1 family also appeared to be divergent in all Tricladida and is the cause of FoxL1/I appearing as an outgroup in the metazoan tree (Fig. 1c). In contrast with the QD family, which was present all along the metazoan tree, these two new subfamilies were only present in the Platyhelminthes superphylum. We have considered them to be Platyhelminthes-specific subfamilies as they still cluster together with their main branch, but do not mix with the other members. In the case of the N(P), it appears to be Triclad-specific. However, a more exhaustive phylogenetic analysis should be performed to support this observation. We propose that the huge losses that took place before the origin of this clade may have caused the duplication and divergence that ultimately lead to the formation of the A(P) and N(P) subfamilies. According to the SCSeq databases both genes are expressed in specific cells populations; *foxA(P)* in ‘activated early epidermal progenitors’ and *foxN(P)* in epidermic, GABAergic and secretory 2 cells, which could agree with its functional specialization after duplication. The RNAi analysis demonstrates they have an important role during regeneration, but further studies are required to investigate their specific role during planarians regeneration.

### Tricladida may have suffered a genome reorganisation

Regardless of the extensive above-mentioned variations in family numbers due to gains and losses, the number of Fox genes present across organisms seems to be constant. The conserved number is roughly 23 genes with some obvious exceptions such as humans or sponges (Fig. 2). This regularity may seem surprising, but as Fernandez and Gabaldon^69^ pointed out, family losses are usually compensated by gene duplications of the remaining family members (as appears to be the case in Platyhelminthes). We cannot discard the possibility that family losses were compensated by the emergence of de novo genes, which could cluster in pre-existing families due to artefacts of the clustering methods^65^. Another interesting aspect to examine is the relation between the number of Fox genes and the number of Fox families. When we study this connection in Platyhelminthes we find the opposite; the number of families remains constant while the number of Fox genes increases in Tricladida (Fig. 4). This could be explained in three ways: i) several tandem duplications occurred before the branching of Tricladida, ii) a partial genome duplication event happened and affected the Fox genes, iii) a whole genome duplication (WGD) event happened. Currently, we do not have enough data to support any of these hypotheses. Other studies that have found gene duplications in Platyhelminthes species propose that WGD events could have occurred^63,64,70,71^. In *Smed*, we found every Fox gene in a different scaffold (except for *foxA1* and *foxD1*) and we could not find any trace of microsynteny retained in the Fox gene regions, not even within the *Smed* genome comparing paralogs-containing scaffolds. It must be noted that although the *Smed* genome is not completely assembled but is still fragmented in 481 scaffolds, most of the scaffolds interrogated were more than 3 Mb long and only two of them shorter than 1 Mb. Thus, in the *Smed* genome we cannot observe any indicator of WGD although a massive reorganization, erasing any trace of it, could have occurred. For clarification, we would need a number of Tricladida available genomes of sufficient quality. Regrettably, the *Smed* genome is the only high quality Tricladida genome currently accessible.

### Specificity of Fox genes families expression and function

The identification of the complete set of Fox genes in *Smed* together with the analysis of the expression pattern and function of the newly identified genes provides a more complete view of the function of each fox gene and family. Some results agree with an evolutionary conserved role of fox families and others do not. For instance, the A family mostly participates in endoderm specification during gastrulation, notochord formation in vertebrates or pharynx formation in cnidarians and planarians^72–76^. Accordingly, in *Smed*, *Djap* and *Spol,* foxA1 is expressed in the pharynx, and particularly in *Smed* it was demonstrated to be fundamental for its specification^33,77,78^. However, we found *foxA2* expression in neuronal cells in de dorsoventral margin and *foxA(P)* in epidermal cells*.* Families C-F-L1-Q1 usually cluster together in the genome and participate in the development of mesoendodermal tissues in many organisms^43,79^. We found no gene cluster arrangement of C-F-L1 families, but those genes might conserve their function determining mesoendodermal tissues in *Smed*, since their expression correlates with muscle cells and, particularly, *foxF2* regulates a specific pharynx muscle type^80,81^. The D family is expressed in notochord and neural crest in chordates, in intestinal precursors in *C.elegans*^82^ and in the nervous system in *Drosophila melanogaster*^83^. In *Smed* and *Djap*, *foxD1* is expressed in anterior muscle cells and participates in the anterior face decision^84–86^. *foxD2* and *foxD3* were found in neurons and muscle cells, respectively. Accordingly, after knocking down *foxD2*, planarians presented strong regenerative defects in the SNC. The J1 family was broadly found in ciliated cells in different animals and tissues^87,88^. In *Smed*, *foxJ1-4* were found expressed in ciliated cells in different epidermal patterns^37^; and the new *foxJ1-5* is found in the epidermis, neuronal and protonephridia cells, suggesting a conserved function of the family in ciliated cells. In vertebrates, the K family has been found expressed in mesodermal and ectodermal tissues^89,90^. In *Smed, foxK1-3* were found in the nervous system and in parenchymal or secretory cells. Interestingly, after the inhibition of three K genes, planarians presented regenerative defects in the nervous system, suggesting that all three genes have maintained a role controlling neurogenesis.

Overall, the integration of the current data regarding Fox families suggests that the expansion of families often results in expression and functional diversification.

### Cell death regulation by *Smed-foxO* is conserved in planarians

Several Fox families have been lost or have been expanded in Platyhelminthes. Interestingly, the FoxO family is one of the few that shows a unique family member. FoxO has crucial roles in controlling molecular process related with aging and cancer^41,69^. FoxO senses oxidative stress and responds through regulating cell motility, stress resistance, or cell death^91^. FoxO also has a conserved role in maintaining cellular energy homeostasis by coordinating cellular supplies and demands^39^. When nutrients are available, InsulinR is activated and FoxO is phosphorylated by AKT, which inhibits its entrance into the nucleus^59,60^. Under conditions of growth factor limitation or other stresses, FoxO enters the nucleus and inhibits mTORC1. Our data shows that *Smed-foxO* is probably regulated by AKT, since it conserves the phosphorylation domains and, furthermore, *Smed-foxO* RNAi inhibition impairs the apoptotic response, which is the opposite phenotype described after AKT RNAi in planarians^77^. Thus, it could be proposed that in starved planarians, the limitation of nutrients inhibits the insulin pathway and AKT, allowing for the increase in levels of unphosphorylated *Smed-foxO* that can enter the nucleus. In the nucleus, *Smed-foxO* activates the apoptotic response required in starved planarians to trigger degrowth^93–98^. In *Smed-foxO* (RNAi) animals this transcriptional activity cannot take place and neither can the apoptotic response. The reduction of cell death after *foxO* inhibition appears to be evolutionarily conserved as it has been also observed in *Hydra*^99^, *C.elegans*^100,101^, *Drosophila melanogaster*^20,102^ and in various mammal tissue types^103–106^. Although the mRNA expression of *foxO* is found in specific cell types its regulation can take place post-transcriptionally^107^, thus with the present data we cannot discern about the contribution of specific tissues to the RNAi phenotype observed in this study. Since we have observed that epidermal cells are disorganized, and this is one of the cell lineages where *foxO* is expressed, we could hypothesize that a decrease in cell death in this cell type could contribute to the phenotype observed.

*Smed-foxO* could also be regulated by other pathways as the Sirtuin family^108^, which senses cellular metabolic state and acetylates FoxO (activation). Recently, Ziman et al. have demonstrated in planarians that upon starvation and after *sirtuin-1* inhibition (*foxO* inhibition) animals display a reduction in cell death^97^.

The cellular REDOX state is not only essential for cellular homeostasis but it is necessary to activate the regenerative response in several models, as well as in planarians^109^. In this study we have shown that *Smed-FoxO* is also required for proper regeneration, but further studies are required to test whether it also senses ROS levels both in regenerating and in intact planarians.

## Conclusions

As we acquire more information on the presence of the TF Fox family across metazoan species, it becomes clearer that some Fox genes originated at the base of metazoans followed by different events of gene loss and diversification as proposed by^42,69,110^. Following this thread, as the number of annotated Fox genes increase, our ability to classify them also improves up until the point where ideally no Fox would be misannotated. In the past, these errors in the annotation led to a misunderstanding of the evolution of conserved functions in different Fox families.

In this study, the new annotation allowed for the proposal of a new family present in most metazoans, the FoxQD, as well as phylum-specific families exclusively found in Platyhelminthes. The appearance of phylum-specific families might not be unique to Platyhelminthes and could have happened several times throughout evolution. To prove this theory, there is a need of a better Fox gene annotation from all across the metazoan species. Besides, the proper phylogeny of these genes is not the only benefit. Having better annotated Fox genes in different key species will also help us to understand how different gene regulatory networks and developmental processes could have evolved.

*Schmidtea mediterranea* is a unique model that raises developmental questions in an evolutionary context due to its position in lophotrochozoans, an under-studied clade, and due to its stem cell-based plasticity. The identification of 27 genes divided into 12 families will give us the bases from which to understand how the TFs take part in the regulation of key molecular pathways that control major developmental roles. In particular, in this study we proved that *Smed-foxO,* which in contrast to other families is constantly present in metazoans, is evolutionary conserved to regulate cell death.

Finally, the identification of complete gene families in *Smed* will also help to understand the evolution of planarians and Platyhelminthes. Here we have seen how in the order Tricladida the number of Fox genes increased while the number of families was retained. However, we could not find traces of neither a genome (whole or partial) duplication event nor tandem duplications of the Fox genes. This indicates that in planarian ancestors a genomic reorganisation could have occurred. A larger amount of Platyhelminthes and Tricladida genomes are needed to clarify these evolutionary scenarios.

## Methods

All methods were carried out in accordance with relevant guidelines and regulations.

Experiments were performed with planarians, flatworms that do not require specific approvals.

### Sequence and phylogenetic analyses

For generating the phylogenetic trees, we first obtained the FOX protein sequences from several different sources. In some of the cases we were able to collect them from the public databases, like in the case of *Hsa* or *Xtr* (S2 Table). For the rest of the organisms a manual annotation was required. If the only resource available was a transcriptome, like in the case of *Tsol*, we used Transdecoder (v5.5.0) to obtain the translated proteins. Using HMMER with default parameters and a cutoff e-value of 1e-4 (v3.1b2) and the Pfam^111^ motive of the Forkhead domain (PF00250.13), we extracted the Forkhead-containing proteins.

The whole set of translated proteins was aligned again using MAFFT^112^ with the L-INS-i strategy and the aligning Forkhead domain was selected. This alignment was the input used for IQ-TREE^113^ to generate the phylogenetic tree. The options used to run the webserver of IQ-TREE were the ones by default, including the automatic substitution model selector and the ultrafast bootstrap analysis, except for the number of bootstrap alignments (set at 2500), the single branch test number of replicates (set at 2000) and the approximate Bayes test option (selected). The trees were visualized using Dendroscope3 v3.6.3^114^ with the default parameters.

For *Smed* FOX domains disposition architecture the NCBI web server was used (http://www.ncbi.nlm.nih.gov/Structure/cdd/wrpsb.cgi) to identify FKH, FHA and FOXP coiled coil;^115^ and^116^ were used to identify NLS and NES, respectively.

### Paralog analysis

The homology relationships between *Smed* and human was assessed with the best hit in a two-way blast (v2.6.0) search against human RefSeq transcripts. For the analysis of *Smed* scaffolds, a blast of the whole genome against the whole genome with the parameters “-evalue 1e-20 -word_size 100” was performed, and then the data was visualized using Circos (v0.69-9), adding a track for repeating elements. Both, the links and the repeats were filtered for only rendering those greater than 1 Kb.

### Animal maintenance

Asexual clonal strain of *Smed* BCN-10 biotype were maintained in PAM water^117^ as previously described^118^. To keep planarian population, animals were fed twice per week with liver, and starved for a week before being used in any experiment.

### Isolation of Fox genes and quantitative real-time PCR

In any experiment, TRIzol reagent (Invitrogen) was used to extract total RNA from intact planarians, and cDNA was synthesized as previously described in^98^. Fox genes PCR fragments were cloned into pGEM-T Easy (Promega) vector for dsRNA synthesis or pCRII (Life Technologies) vector to ssRNA synthesis. Nucleotide sequence data reported are available in the Third Party Annotation Section of the DDBJ/ENA/GenBank databases under the accession numbers TPA: BK010973-BK010987. Quantitative real-time PCRs were performed on 7500 Fast PCR System (Applied Biosystems), experiments were conducted using 3 biological and 3 technical replicates for each condition. Expression levels were normalized to that of the housekeeping gene ura4. All primers used in this study are shown at S6 File.

### Whole-mount in situ hybridization

SP6 or T7 polymerase and DIG- or FITC- modified (Roche) were used to synthesise RNA probes in vitro. For colorimetric whole-mount in situ hybridization (WISH) the previously described^119^ protocol was followed. Animals were sacrificed with 5% *N*-acetyl-l-cysteine (NAC), fixed with 4% formaldehyde (FA), and permeabilized with Reduction Solution.

### RNAi experiments

Double strand RNA (dsRNA) was synthesised by in vitro transcription (Roche) as previously described^120^. Injections of dsRNA (3 × 32.2 nl) were carried into the digestive system of each animal on 3 consecutive days per week using Nanoject II injector (Drummond Scientific Company). Experiments in intact animals were conducted with starved animals undergoing 2 consecutive weeks of injection, without amputation. In regenerating experiments, animals underwent two weeks of inhibition and amputation. *gfp* was injected as a control.

### Immunohistochemistry staining

Whole-mount immunohistochemistry staining was performed as previously described^121^. Animals were sacrificed with 2% HCl and fixed with 4% FA. Animals were blocked in 1% bovine serum albumin (BSA) in 1X PBSTx 0,3% (Blocking Solution) for 2 h at RT. Primary antibodies were diluted in blocking solution and incubated 16 h rocking at 4 °C. Then, washes were per performed for at least 4 h. Secondary antibodies were diluted in blocking solution for 16 h rocking at 4 °C.

The following antibodies were used in these experiments: mouse anti-synapsin (anti-SYNORF1, 1:50; Developmental Studies Hybridoma Bank, Iowa City, IA, USA), rabbit anti-phospho-histone H3 (Ser10) (D2C8) (PH3) (1:500; Cell Signaling Technology) and mouse anti-VC1 (anti-arrestin, 1:15,000, kindly provided by Professor K. Watanabe). The secondary antibody used was Alexa 488-conjugated goat anti-mouse (1:400; Molecular Probes, Waltham, MA, USA) and Alexa 568-conjugated goat anti-rabbit (1:1000: Molecular Probes, Waltham, MA, USA). Nuclei were stained with DAPI (1:5000; Sigma).

### Caspase 3 activity assay

At the end of the second week of RNAi inhibition, protein extraction was performed. BioRad protein reagent was used to obtain protein concentration of the cell lysates. Fluorometric analysis of caspase-3 activity was performed as described previously^122^. Fluorescence was measured in a luminescence spectrophotometer (Perkin- Elmer LS-50) using Fluostar Optima microplate fluorescence reader (BMG Labtech), applying the following settings: excitation, 380 nm; emission, 440 nm. 20 mg of protein extract was used to determine enzyme activity, incubating for 2 h at 37 °C with 20 µM caspase-3 substrate Ac-DEVD-AMC or 2 ml from a stock of 1 mg/ml for a final volume of 150 µl. Three technical replicates were analysed per condition.

### TUNEL assay on paraffin sections

Animals were sacrificed with 2%HCl and fixed with 4%PFA. Paraffin embedding and sectioning were carried out as previously described in^123^. Slides were de-waxed and re-hydrated as previously described in^124^. Sections were treated as described previously in^125^ and after the dewaxing step, they were incubated with Proteinase K (20 µg/ml for 10 min at room temperature). Finally, the ApopTag Red In situ Apoptosis Detection Kit (CHEMICON, S7165) was used, following manufacturer’s protocol. Nuclei were stained with DAPI (1:5000; Sigma).

### Imaging and quantification

WISH images were captured with a ProgRes C3 camera from Jenoptik (Jena, TH, Germany). In vivo images were obtained using Scmex 3.0 camera in a Zeiss Stemi SV 6 binocular loupe and measured suing Fiji Y. A Zeiss LSM 880 confocal microscope (Zeiss, Oberkochen, Germany) was used to obtain confocal images of whole-mount immunostainings, TUNEL staining and epidermal sections. Representative confocal stacks for each experimental condition are shown. Cell counting of PH3 + and TUNEL staining was carried out by eye quantification in a previous defined area of each animal. Areas are schematically indicated in each figure. The total number of PH3 + cells was divided by the animal area. For TUNEL quantification, TUNEL positive cells were counted in at least 30 representative transversal sections per animal. The number of positive cells were divided by the mean area of the all sections in each animal. For epidermal cell density, the number of nuclei were manually counted and divided per the total image area. Images were blind analysed and later grouped according to each genotype. At least two animals were analysed per condition.

### Statistical analysis and presentation

Statistical and presentation analyses were performed using GraphPad Prism 8. Two-sided Student’s t-tests (α = 0.05) were performed to compare the means of 2 populations. To compare 2 populations, we used box plots depicting the median, the 25th and 75th percentiles (box), and all included data points (black dots). Whiskers extend to the largest data point within the 1.5 interquartile range of the upper quartile and to the smallest data point within the 1.5 interquartile lower ranges of the quartile.

## Supplementary Information

Supplementary Information 1.

Supplementary Information 2.

Supplementary Information 3.

Supplementary Information 4.

Supplementary Information 5.

Supplementary Information 6.

Supplementary Information 7.

Supplementary Information 8.

Supplementary Information 9.

## Abbreviations

*Amq*: *Amphimedon queenslandica*
*Bla*: *Branchiostoma lanceolatum*
*Bose*: *Bothrioplana semperi*
*Cate*: *Catenulia*
*Cgi*: *Crassostrea gigas*
*Cte*: *Capitella teleta*
*Djap*: *Dugesia japonica*
*Dlac*: *Dendrocoelum lacteum*
*Dme*: *Drosophila melanogaster*
*Emul*: *Echinoccocus multiocularis*
FHA: Forkhead associated domain
FKH: Forkhead domain
Fox: Forkhead Box
*Geap*: *Geocentrophora applanta*
*Gsa*: *Gyrodactylus salaris*
*Hro*: *Helobdella robusta*
*Hsa*: *Homo sapiens*
*Ili*: *Intoshia linei*
*Lept*: *Leptoplana tremellaris*
*Lgi*: *Lottia gigantea*
*Lna*: *Lingula anatine*
*Meli*: *Mesostoma lingua*
*Mli*: *Macrostomum lignano*
*Mli*: *Macrostumum lignano*
*Mosp*: Monocelis sp.
NES: Nuclear exportation signal
NLS: Nuclear localization signal
*Nve*: *Nematostella vectensis*
*Obi*: *Octopus bimaculoides*
*Pfl*: *Ptychodera flava*
*Pnig*: *Polycelis nigra*
*Pten*: *Polycelis tenuis*
*Ptor*: *Planaria torva*
*Sdo*: *Suberites domuncula*
*Sko*: *Saccoglossus kowalevskii*
*Sman*: *Schistosoma mansoni*
*Smed*: *Schmidtea mediterranea* Asexual strain
*Smes*: *Schmidtea mediterranea* Sexual strain
*Spol*: *Schmidtea polychroa*
*Spu*: *Strongylocentrotus purpuratus*
*Tca*: *Tribolium castaneum*
TF: Transcription Factor
*Tsol*: *Taenia solium*
WGD: Whole Genome Duplication
*Xtr*: *Xenopus tropicalis*
