# Supplementary material for: Analysis of Fox genes in Schmidtea mediterranea reveals new families and a conserved role of Smed-foxO in controlling cell death
Source: Sci Rep. 2021 Feb 3;11:2947. doi: 10.1038/s41598-020-80627-0 (PMC7859237; doi:10.1038/s41598-020-80627-0)
Supplement: Supplementary file 9 — Supplementary Information 9. [file 41598_2020_80627_MOESM9_ESM.pdf]

Analysis of Fox genes in *Schmidtea mediterranea* reveals new families and a conserved role of *Smed-foxO* in controlling cell death.

Eudald Pascual-Carreras [(1) (2) +*], Carlos Herrera-Úbeda [(1) (2) +], Maria Rosselló [(1) (2)], Pablo Coronel-Córdoba [(1) (2)], Jordi Garcia-Fernandez [(1) (2)], Emili Saló [(1) (2)] & Teresa Adell [(1) (2) *]

[(1)] Department of Genetics, Microbiology and Statistics and Institute of Biomedicine, Universitat de Barcelona, Barcelona, Catalunya, Spain.

[(2)] Institut de Biomedicina de la Universitat de Barcelona (IBUB), Universitat de Barcelona, Barcelona, Catalunya, Spain.

The PDF file includes:

Figs. S1 to S13

Other Supplementary information of this manuscript includes the following:

S1 File

In this file we have compiled all the data used for generating the phylogenetic tree of Metazoa. Alongside the internal identificator used in the trees, there is the original ID, the database from where the sequences were retrieved, the species, the domain sequence and the complete protein sequence. We have also added the number of families, and genes for each of the species analysed. If the source of the data was obtained from a publication, the field is filled with the reference.

S2 File

Raw tree of phylogenetic Fox analysis from Metazoa species, placed in Fig. 1b.

S3 File

In this file we have compiled all the data used for generating the phylogenetic tree of Platyhelmintes. Alongside the internal identificator used in the trees, there is the original ID, the database from where the sequences were retrieved, the species, the domain sequence and the complete protein sequence. We have also added the number of families, and genes for each of the species analysed. If the source of the data was obtained from a publication, the field is filled with the reference.

S4 File

Raw tree of phylogenetic Fox analysis from Plathelmintes species, placed in Fig. 3a.

S5 File

In this file we can find the position of every Fox gene identified in *Smed* along with a summary of the microsyntenic features found by Irimia et al. [1] with a Fox gene involved separated by family. We have also compiled the three upstream and downstream annotated orthologs (by best hit against human; * indicates orthologies with two-way BLAST hits) for every Fox gene in *Smed*, as well as the three upstream and downstream coding genes for each Fox gene in *Homo sapiens* whose family is present in *Smed*. Finally in a separate tab is the full list of *Hsa* Fox genes with their three upstream and downstream neighbours. Orthologies are indicated by same coloured fonts.

S6 File. Primers used in this study

We display: name of the primer, technique where they used and the sequences forward (Fw) and reverse (Rv) 5ôto 3ô

S1 Table. Fox genes in *Schmidtea mediterranea*. The following information is shown: genome ID and its scaffold disposition, the transcriptome ID [2]; new and previous names of Fox genes in *Smed*; GeneBank IDs of new and previous *Smed* Fox genes; the homologs identified in relative planarian species and the reference where they were mentioned before; the RNAi phenotype observed after its inhibition and the references where they were mentioned before; the expression pattern obtained by WISH and from the SCseq databases; and the references corresponding to the Fox already analyzed by WISH. N.P. ï No Phenotype / N.D. no detected / N.S.C. No Specific Cluster

S2 Table. Fox protein sources. Fox protein sources. For each species, the source of their Fox genes and the database or the previous work is indicated: Larroux C et al (2008) [3], Marletaz F et al. (2018) [4], Egger B et al. (2015) [5], Wang J et al. (2012) [6], Simakov O et al. (2013) [7], Rozanski A et al. (2019) [2], Clark A et al. (2007) [8], Church DM et al. (2011) [9], Mikhailov KV et al. (2016) [10], Luo YJ et al. (2015) [11], Putnam NH et al. (2007) [12], Zarrella I et al. (2019) [13], Simakov O et al. (2015) [14], Adell T et al. (2004) [15], Grohme M et al. (2018) [16], Sodergren E et al. (2006) [17], Richards S et al. (2008) [18] and Hellsten U et al. (2010) [19].

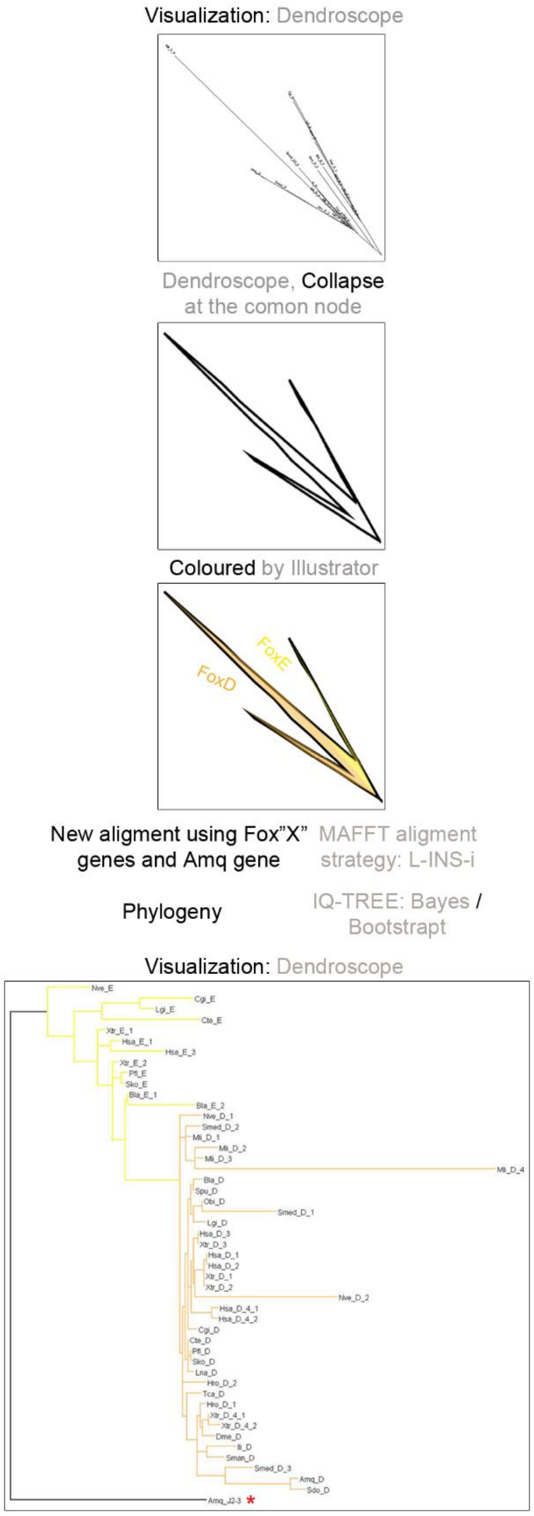

Figure S1. Family tree representation workflow. Families are collapsed at the common node and schematic colour was added. All genes per family were collected, aligned and a new phylogenetic analysis was done. All branches belonging to the same family are coloured equal to the family represented in the main tree. To obtain a better visualization, an

Amq gene was used as a root (red asterisk): *Amq-foxJ2/3* for Clade I and *Amq-foxD* for Clade II genes.

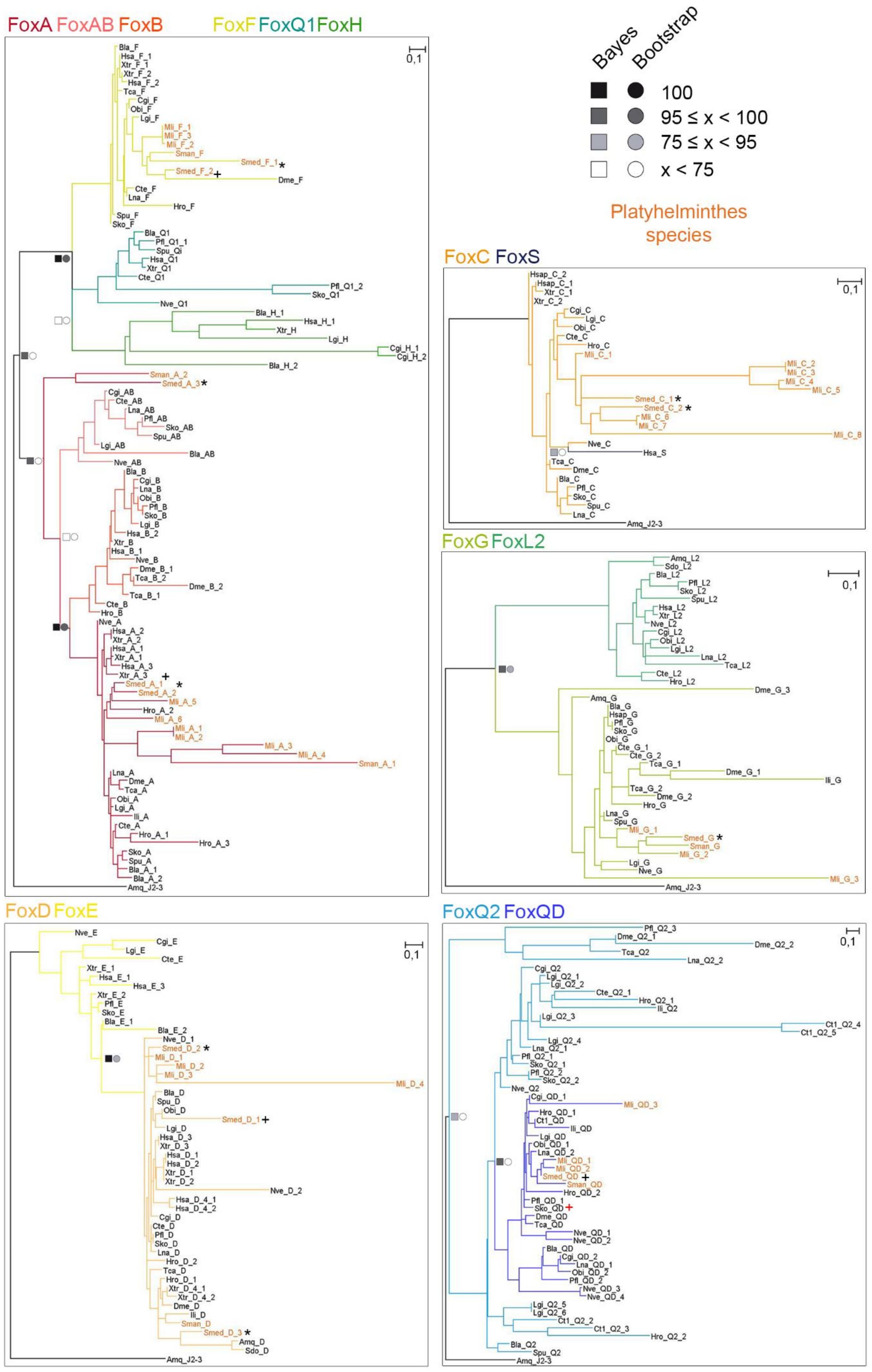
FoxA FoxAB FoxB
FoxF FoxQ1 FoxH
Bayes
Bootstrap
100
95 ≤ x < 100
75 ≤ x < 95
x < 75
Platyhelminthes species
FoxC FoxS
FoxG FoxL2
FoxD FoxE
FoxQ2 FoxQD
0,1

Figure S2. Phylogenetic trees from node-sharing families. The ML phylogenetic trees based on the FKH domain. At nodes, values for the approximate Bayes (square) and Likelihood (circle) ratio test are shown. Colour indicates % of confidence. For each node-sharing families, phylogenetic trees were created using an *Amq* gene from the opposite clade as out group. Family branches are painted with the same colour as they are represented in the main tree. Platyhelminthes genes are coloured light orange. Dark cross indicates previous characterized gene and dark asterisk indicates new fox characterized in *Schmidtea mediterranea* (*Smed*). Red cross indicates *Saccoglossus kowalewski foxQD* gene. Aminoacidic sequences used are found in Additional File 1. Scale indicates expected aminoacidic substitution per site. Species used are the following ones: *Homo sapiens* (*Hsa*), *Xenopus tropicalis* (*Xtr*), *Branchiostoma lanceolatum* (*Bla*), *Strongylocentrotus purpuratus* (*Spu*), *Saccoglossus kowalewski (Sko*) and *Ptychodera flava* (*Pfl*), *Drosophila melanogaster* (*Dme*), *Tribolium castaneum* (*Tca*), *Crassostrea gigas* (*Cgi*), *Lottia gigantea* (*Lgi*), *Octopus bimaculoides* (*Obi*), *Lingula anatina* (*Lna*), *Intoshia linei* (*Ili*), *Capitella teleta* (*Cte*), *Helobdella robusta* (*Hro*), *Macrostomum lignano* (*Mli*), *Schistosoma mansoni* (*Sman*), *Nematostella vectensis* (*Nve*), *Amphimedon queenslandica* (*Amq*) and *Suberites domuncula* (*Sdo*).

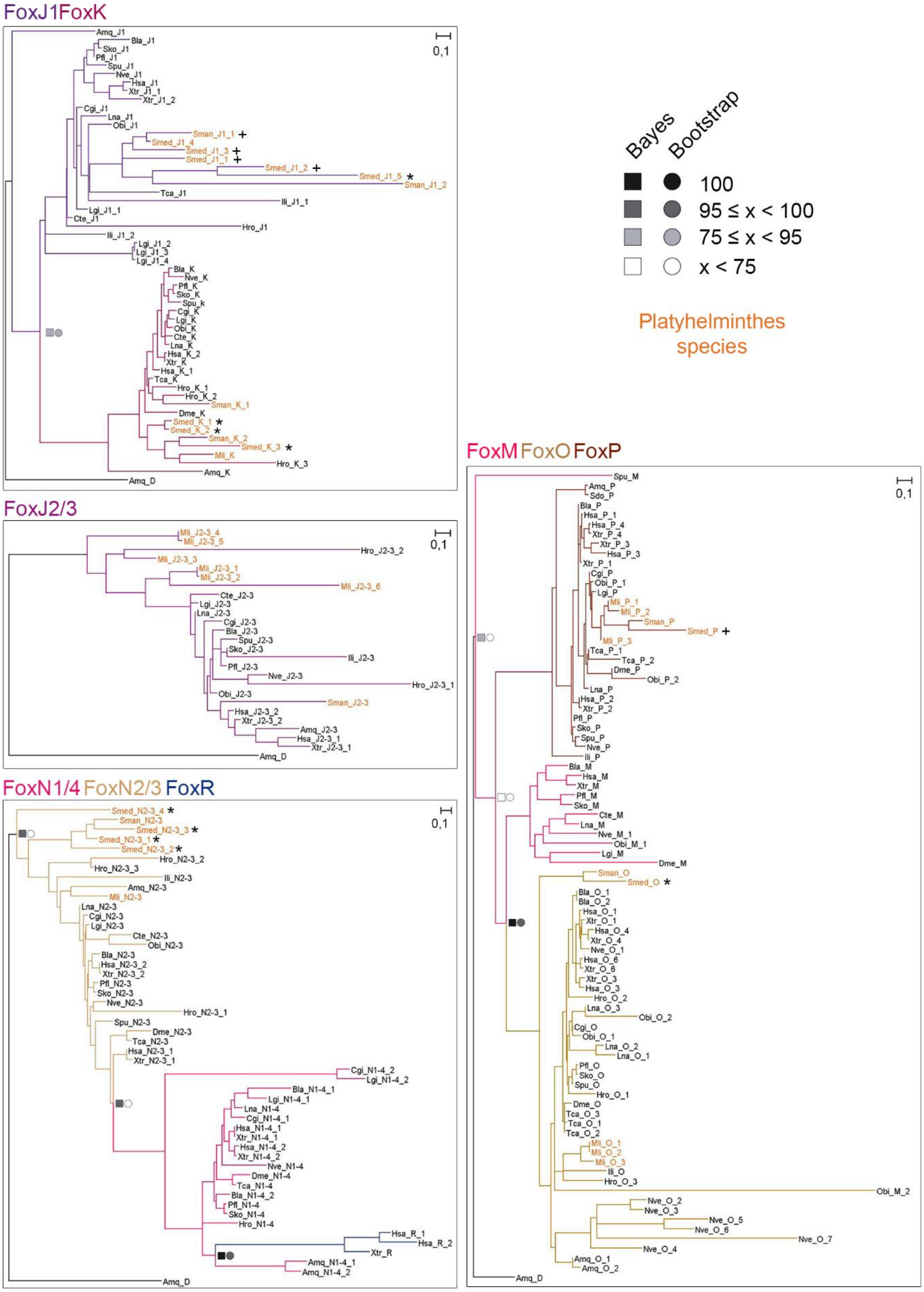

Figure S3. Phylogenetic trees from node-sharing families. The ML phylogenetic trees based on the FKH domain. At nodes, values for the approximate Bayes (square) and Likelihood (circle) ratio test are shown. Colour indicates % of confidence. For each node-sharing families, phylogenetic trees were created using an *Amq* gene from the opposite

clade as out group. Family branches are painted with the same colour as they are represented in the main tree. Platyhelminthes genes are coloured light orange. Dark cross indicates previous characterized gene and dark asterisk indicates new fox characterized in *Schmidtea mediterranea* (*Smed*). Aminoacidic sequences used are found in Additional File 1. Scale indicates expected aminoacidic substitution per site. Species used are the following ones: *Homo sapiens* (*Hsa*), *Xenopus tropicalis* (*Xtr*), *Branchiostoma lanceolatum* (*Bla*), *Strongylocentrotus purpuratus* (*Spu*), *Saccoglossus kowalewski (Sko*) and *Ptychodera flava* (*Pfl*), *Drosophila melanogaster* (*Dme*), *Tribolium castaneum* (*Tca*), *Crassostrea gigas* (*Cgi*), *Lottia gigantea* (*Lgi*), *Octopus bimaculoides* (*Obi*), *Lingula anatina* (*Lna*), *Intoshia linei* (*Ili*), *Capitella teleta* (*Cte*), *Helobdella robusta* (*Hro*), *Macrostomum lignano* (*Mli*), *Schistosoma mansoni* (*Sman*), *Nematostella vectensis* (*Nve*), *Amphimedon queenslandica* (*Amq*) and *Suberites domuncula* (*Sdo*).

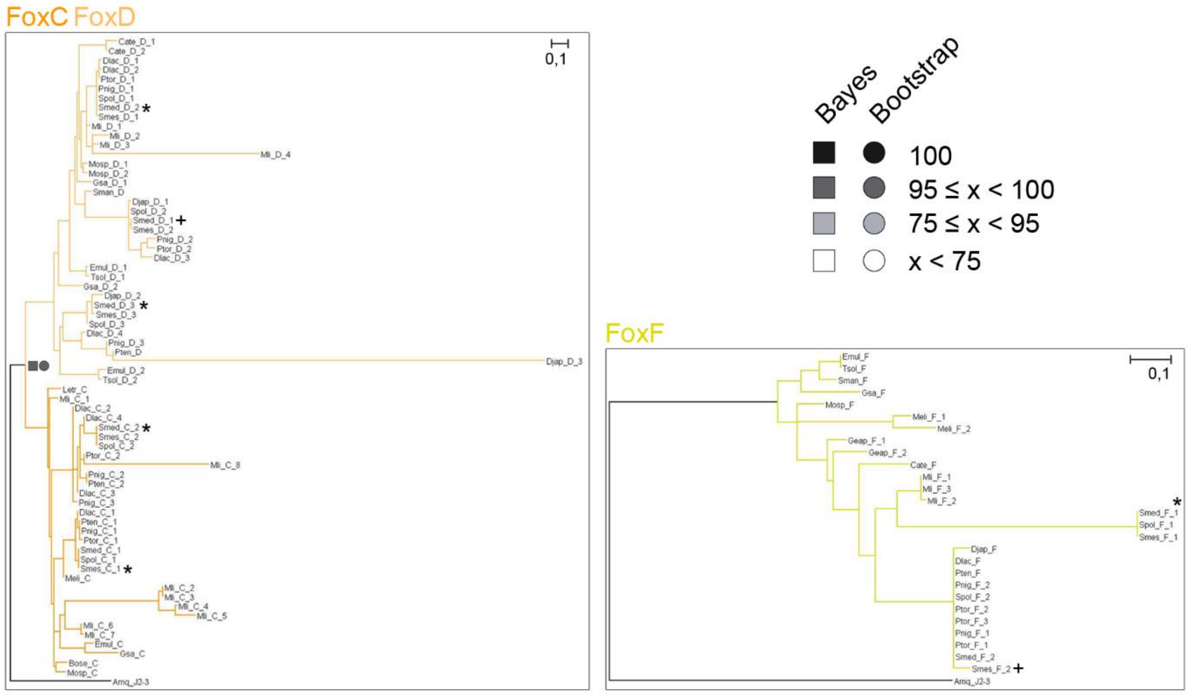

Figure S4. Phylogenetic trees from node-sharing families. The ML phylogenetic trees based on the FKH domain. At nodes, values for the approximate Bayes (square) and Likelihood (circle) ratio test are shown. Colour indicates % of confidence. For each node-sharing families, phylogenetic trees were created using an *Amq* gene from the opposite clade as out group. Family branches are painted with the same colour as they are represented in the main tree. Dark cross indicates previous characterized gene and dark asterisk indicates new fox characterized in *Schmidtea mediterranea* (*Smed*). Aminoacidic sequences used are found in Additional File 3. Scale indicates expected aminoacidic substitution per site. Species used are the following ones: *Taenia solium* (*Tsol*), *Echinococcus multilocularis* (*Emul*), *Gyrodactylus salaris* (*Gsa*), *Bothrioplana semperi* (*Bose*), *Macrostomum lignano* (*Mli*), *Monocelis sp.*(*Mosp*), *Mesostoma lingua* (*Meli*), *Leptoplana tremellaris* (*Lept*), *Geocentrophora applanta* (*Geap*), *Catenulia* (*Cate*), *Planaria torva* (*Ptor*), *Polycelis nigra* (*Pnig*), *Polycelis tenuis* (*Pten*), *Dendrocoelum lacteum* (*Dlac*), *Dugesia japonica* (*Djap*), the sexual strain of *Schmidtea mediterranea* (*Smes*) and *Schmidtea polychroa* (*Spol*).

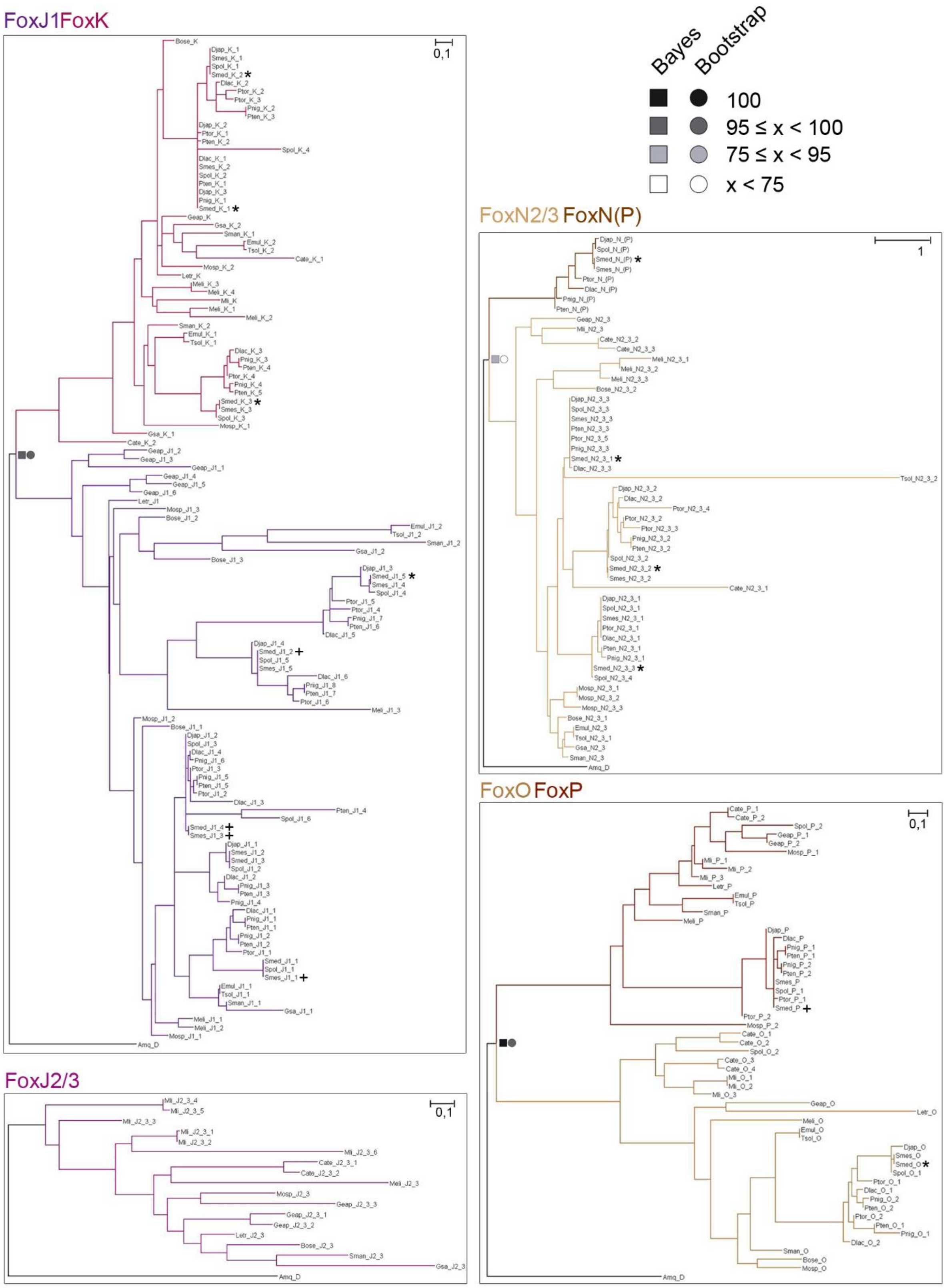

Figure S5. Phylogenetic trees from node-sharing families. The ML phylogenetic trees based on the FKH domain. At nodes, values for the approximate Bayes (square) and Likelihood (circle) ratio test are shown. Colour indicates % of confidence. For each node-

sharing families, phylogenetic trees were created using an *Amq* gene from the opposite clade as out group. Family branches are painted with the same colour as they are represented in the main tree. Dark cross indicates previous characterized gene and dark asterisk indicates new fox characterized in *Schmidtea mediterranea* (*Smed*). Aminoacidic sequences used are found in Additional File 3. Scale indicates expected aminoacidic substitution per site. Species used are the following ones: *Taenia solium* (*Tsol*), *Echinococcus multilocularis* (*Emul*), *Gyrodactylus salaris* (*Gsa*), *Bothrioplana semperi* (*Bose*), *Macrostomum lignano* (*Mli*), *Monocelis sp.*(*Mosp*), *Mesostoma lingua* (*Meli*), *Leptoplana tremellaris* (*Lept*), *Geocentrophora applanta* (*Geap*), *Catenulia* (*Cate*), *Planaria torva* (*Ptor*), *Polycelis nigra* (*Pnig*), *Polycelis tenuis* (*Pten*)*, Dendrocoelum lacteum* (*Dlac*), *Dugesia japonica* (*Djap*), the sexual strain of *Schmidtea mediterranea* (*Smes*) and *Schmidtea polychroa* (*Spol*).

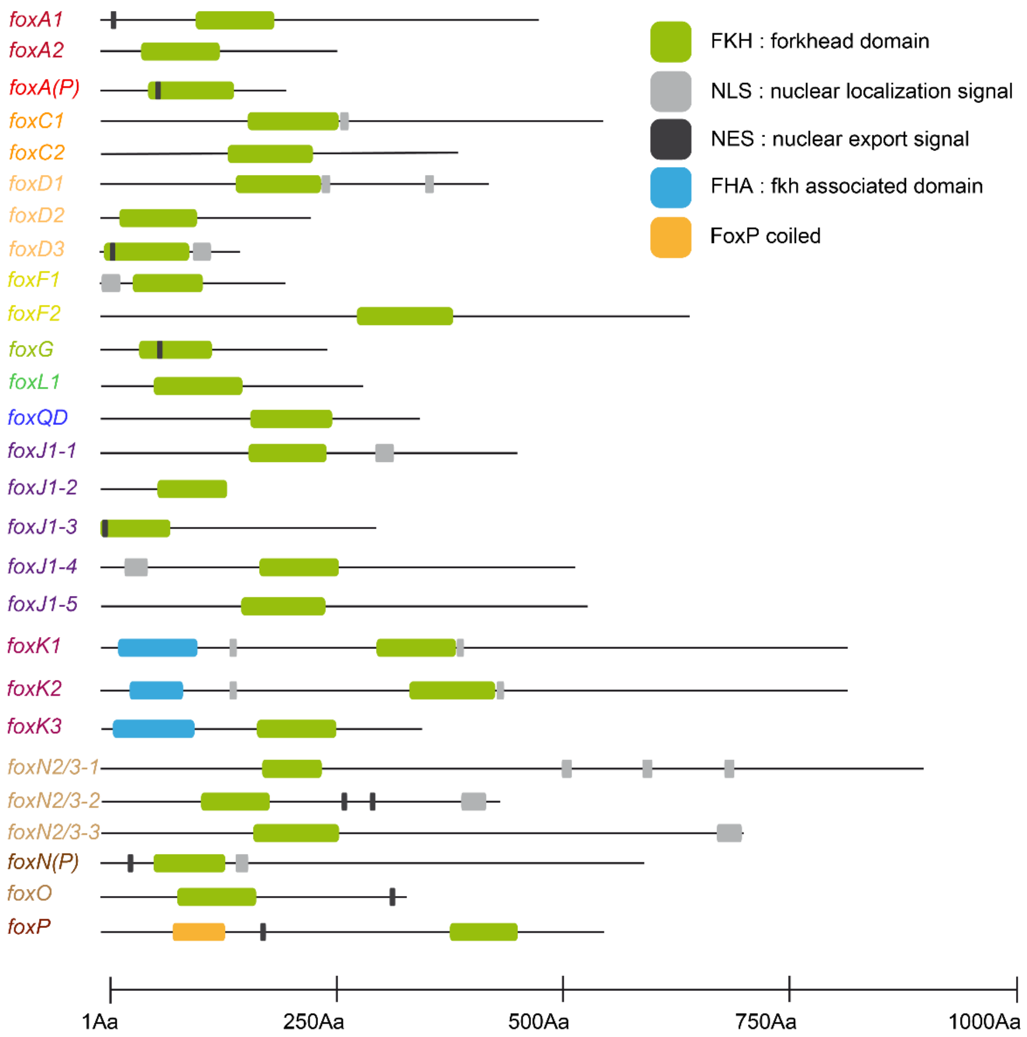

Figure S6. Domains of Fox proteins in *Schmidtea mediterranea*. Conserved domains in planarians: Forkhead domain (green), Forkhead associated domain (blue), FoxP coiled (yellow), nuclear localization signal (light grey) and nuclear export signal (dark grey).

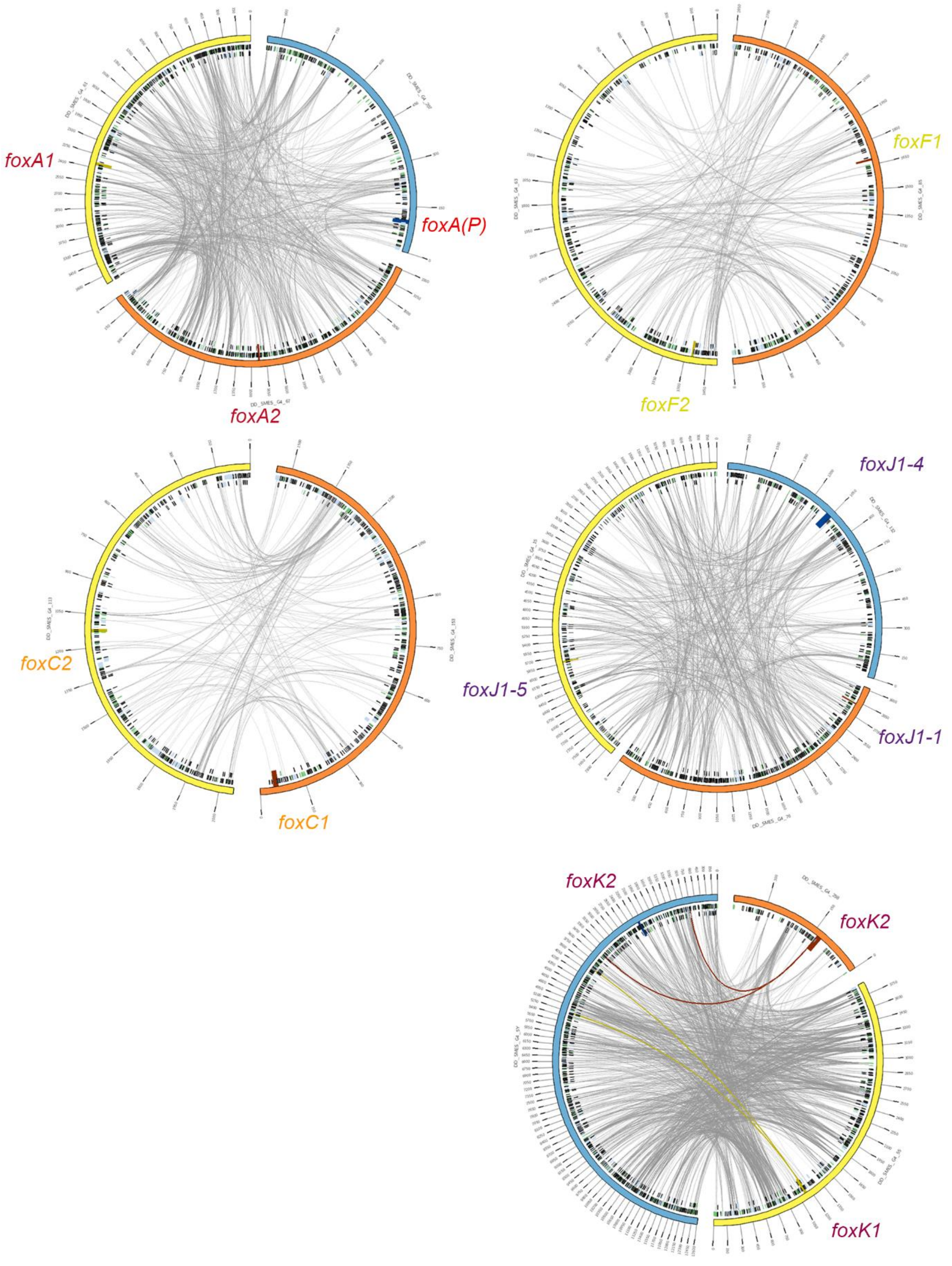

Figure S7. *Smed* genomic alignments between scaffolds containing same-family Fox genes represented with Circos. The Circos representation is composed of two tracks: In the outer ring, the scaffolds containing Fox genes are labelled with their name and in the

outer ring (each tick representing 150kb); in the inner ring, the repeating elements [2] coloured in green (LINEs), blue (TLR) and black (simple repeats and other). Repeats are filtered to be shown only when greater than 1kb. Grey lines connecting the scaffolds are the representation of the alignments, filtered to be shown only when greater than 1Kb. In each scaffold, the region corresponding to the Fox gene (+-5Kb) is represented as a perpendicular darker region, and all the links that fall onto it are coloured accordingly.

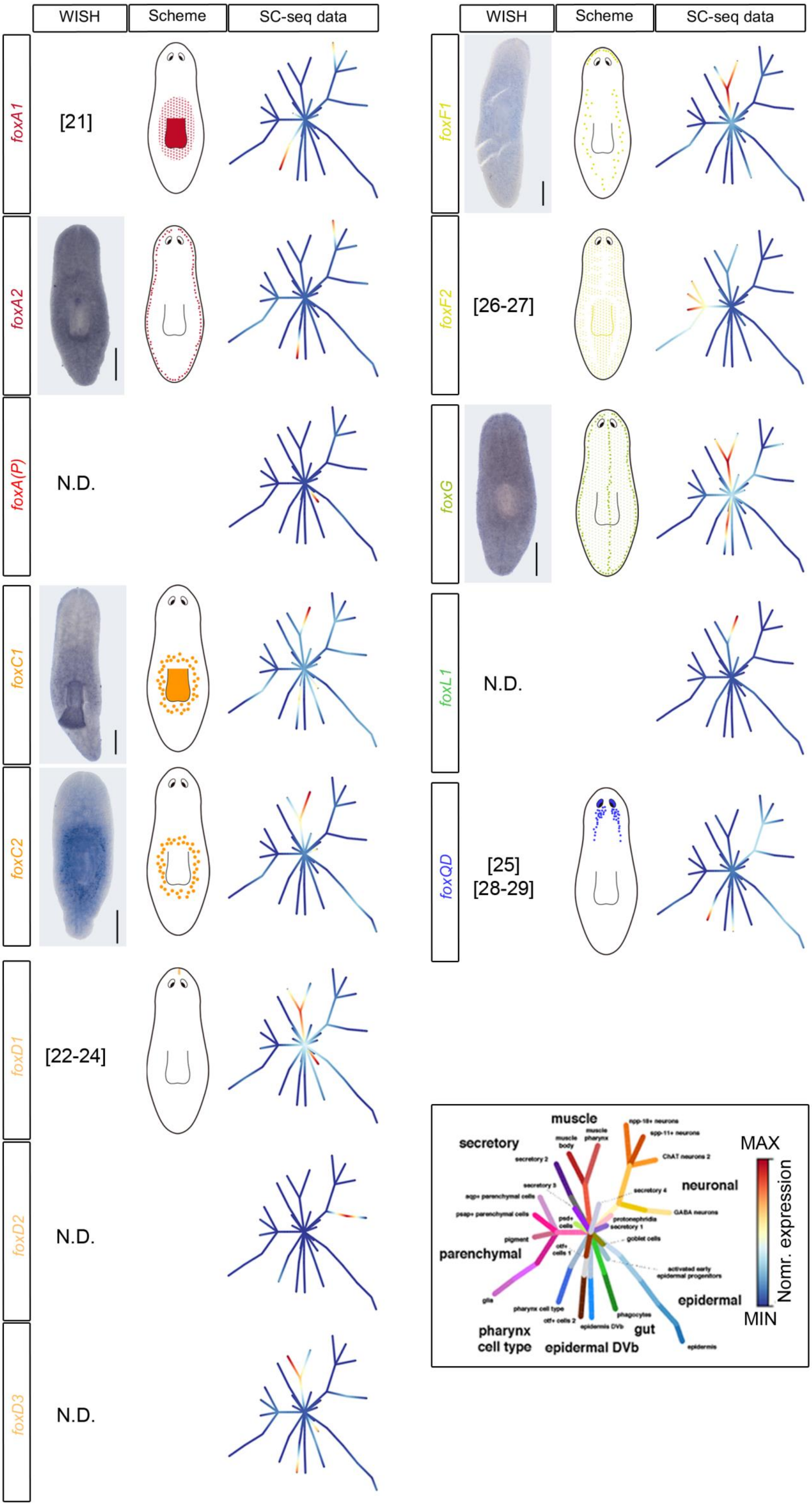

WISH
Scheme
SC-seq data
foxA1
[21]
foxA2
foxA(P)
N.D.
foxC1
foxC2
foxD1
[22-24]
foxD2
N.D.
foxD3
N.D.
WISH
Scheme
SC-seq data
foxF1
foxF2
[26-27]
foxG
foxL1
N.D.
foxQD
[25]
[28-29]
muscle
secretory
neuronal
parenchymal
epidermal
pharynx cell type
epidermal DVb
gut
muscle body
muscle pharynx
secretory 2
secretory 3
secretory 4
secretory 1
ChAT neurons 2
GABA neurons
protonephridia
goblet cells
pigment
glia
pharynx cell type
epidermis DVb
phagocytes
epidermis
activated early epidermal progenitors
MAX
MIN
Nomr. expression

Figure S8. Clade I *Smed-fox* genes show specific expression patterns. WISH of Fox genes in planarians. The expression of *foxA(P), D2, D3* and *L1* genes was not possible to be detected, although at least two different riboprobes were designed. For all genes a schematic cartoon showing expression is added. Graphical representation of *Smed-fox* genes expression during cell differentiation obtained from Plass et al. is shown [20]. Within the A family, *foxA1* was expressed in the pharynx (yellow dashed square) and its progenitors [21] and in both SCSseq was also found in pharynx cell type or in neurons specifically presented in this organ; *foxA2* was marginally expressed in a dotted pattern all along the animal body SCSseq database from Plass et al. revealed that those positive cells could be neurons and/or epidermal cells; the new *foxA(P)* could not be detected, but the SCSeq data suggests that it could be expressed in early epidermal progenitors and/or non-ciliated neurons. ISH revealed that both *foxC* genes were expressed around the pharynx. *foxC2* was also expressed at the pharynx itself. The SCSseq data sets show that those cells could be neural, epidermal or muscle cells, particularly related with the pharynx. *foxD1* was expressed in anterior muscle cells [22ï 24]. The other two D family genes were not detected by ISH. However, the SCSeq data revealed that *foxD2* could be present in some neural progenitors and non-ciliated neuronal cell types. Interestingly, *foxD3* was found at different muscle cell types, early epidermal progenitors and neurons otf+2 (Fig. S7). One member of the F family, *foxF1* was expressed in cells in the margin of the head and in the lateral dorsal part of the animal, between the pharynx and the margin of the organism [25], SCSseq database validated that they were muscle cells. *foxF2*, was previously described to be broadly expressed in the parenchyma embedding all the tissues, in specific cell types: muscle (non-body wall) and pigment cells [26,27]. *foxG* was expressed in a subset of muscle and otf+ neuronal cells all along the DV margin, in the dorsal midline and some scattered cells in the dorsal and ventral part. *foxL1* was not detected by ISH. The SCSeq data indicates that it could be found in a muscular pharynx cell type (Fig. S7). *foxQ/D* was expressed in differentiated eye cells (rhabdomeric photoreceptor neurons), some brain progenitors and in ventral nerve cords [25,28,29]. Additionally, the SCSseq data set from Fincher et al corroborated its presence in a subset of non-ciliated neurons. Scale bars: 250 µm.

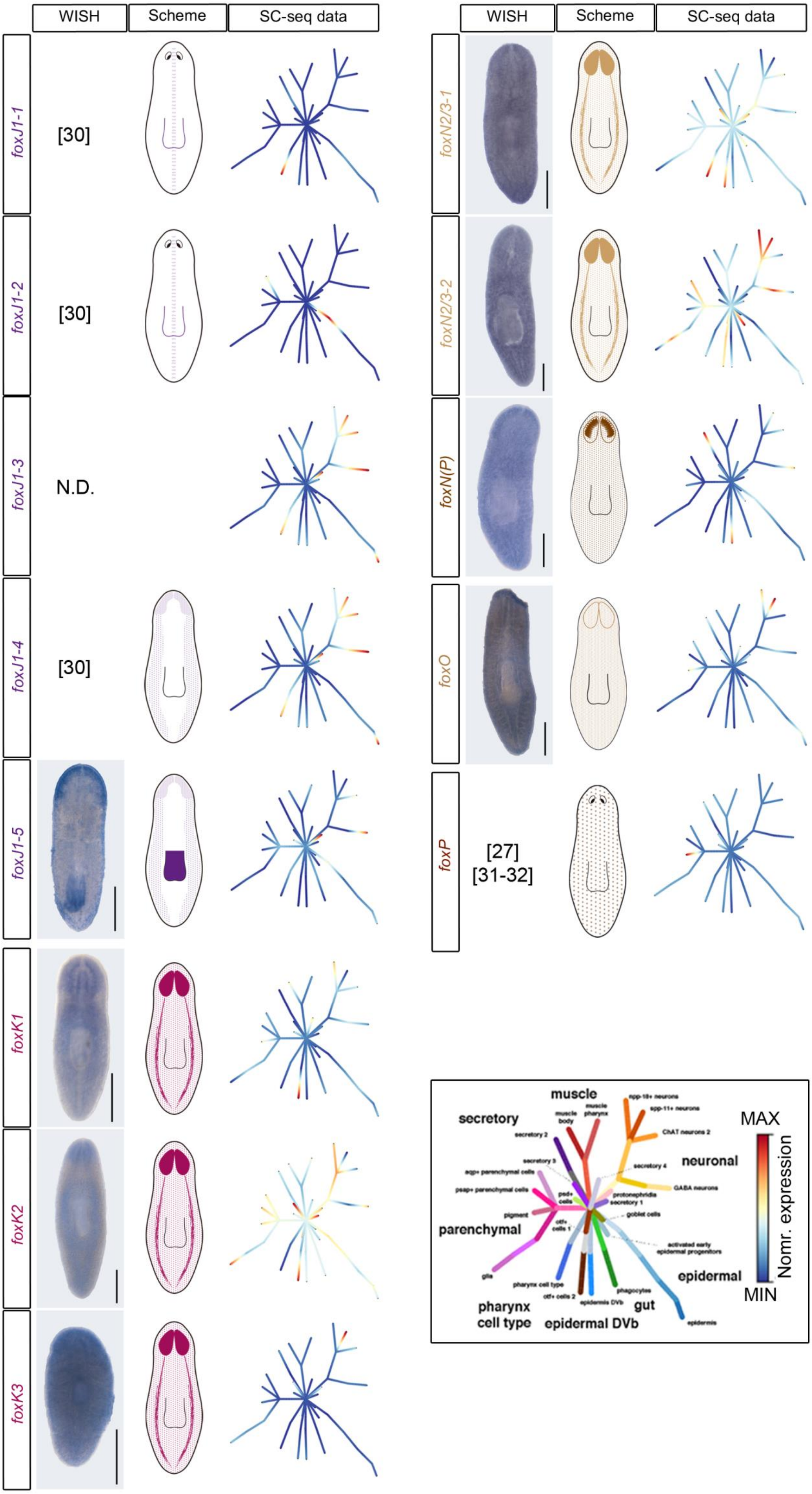

WISH
Scheme
SC-seq data
foxJ1-1
[30]
foxJ1-2
[30]
foxJ1-3
N.D.
foxJ1-4
[30]
foxJ1-5
foxK1
foxK2
foxK3
foxN2/3-1
foxN2/3-2
foxN(P)
foxO
foxP
[27]
[31-32]
muscle
secretory
neuronal
parenchymal
epidermal
pharynx cell type
epidermal DVb
gut
MAX
MIN
Nomr. expression

Figure S9. Clade II *Smed-fox* genes show specific expression patterns. WISH of Fox genes in planarians. The expression of some Fox genes was not possible to be detected, although at least two different riboprobes were designed. For all genes a schematic cartoon showing expression is added. Graphical representation of *Smed-fox* genes expression during cell differentiation obtained from Plass et al. is added [20]. Three J1 family paralogs previously described were expressed in ciliated cells. They disposition could be more dorsally or more ventrally depending on the gene [30], being associated to different cell types by the SCSseq, such as: pharynx, muscle, neuronal and epidermis The non-previously described *foxJ1-5* was also expressed in the epidermis more concentrated in the head area and in the pharynx. This expression was associated with a neural cell type thanks to the SCSseq. Within the K family, *foxK1*, *foxK2* [31] and *foxK3* genes were expressed ubiquitously and specifically in the CNS. The SCSseq verified that these genes were overrepresented in different cell types, such as: parenchymal, secretory and neuronal. The N family contains 4 genes: *foxN2/3-1* [31] and *foxN2/3-2* were expressed ubiquitously and were also expressed in the SNC; *foxN2/3-3* was not detected by ISH; *foxN(P)* was expressed at the edge of the brain, in the brain branches. The SCSseq dataset confirmed their major expression in neural cell type but also in other ones, such as: secretory, parenchymal and epidermal. *foxO* was expressed ubiquitously [31], and SCSeq reveals specific expression in some neural, secretory, epidermal and parenchymal populations. *foxP* is expressed in a specific parenchymal cell type, pigment cells [27,31,32]. Scale bars: 250 µm.

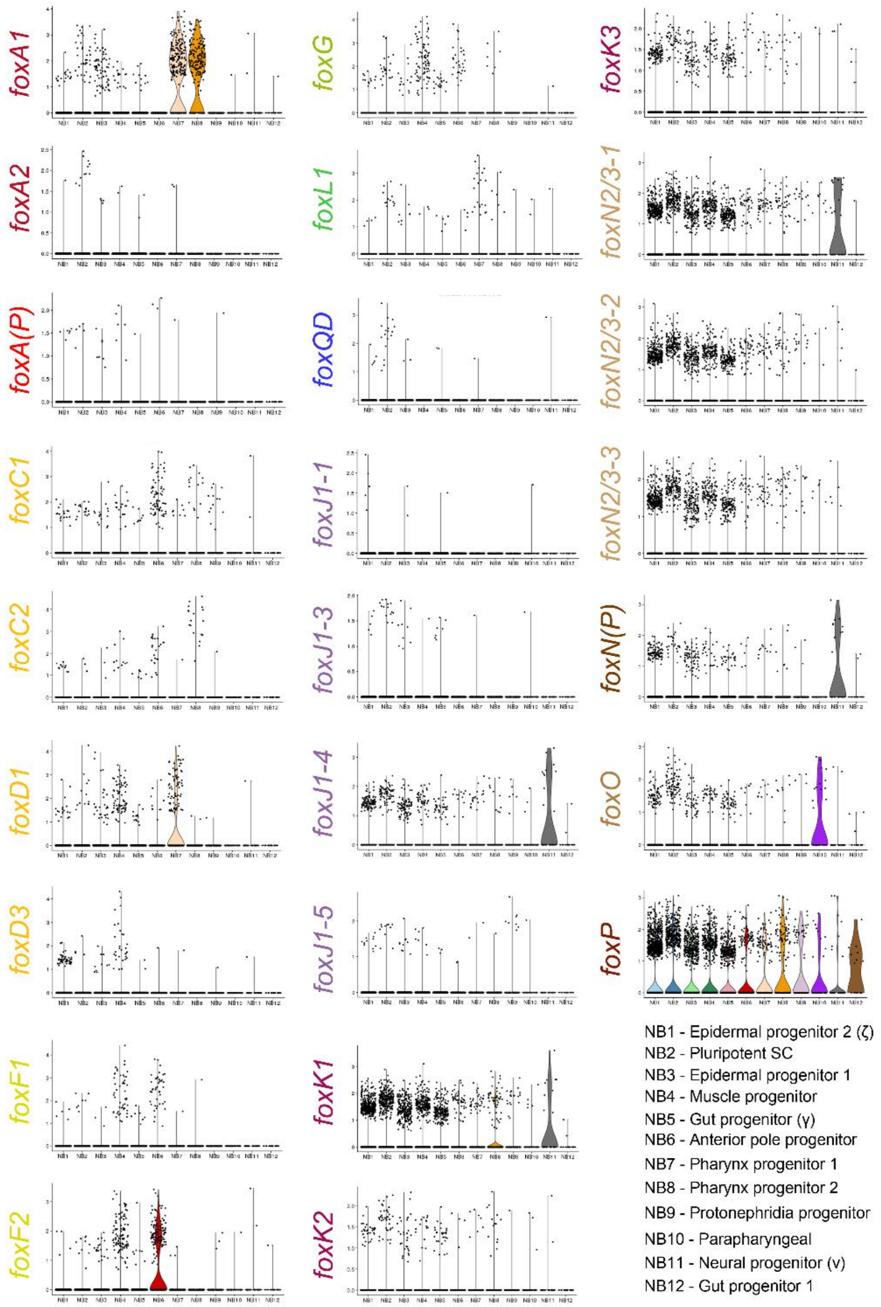

Figure S10. Graphical representation of *Smed-fox* genes expression in neoblasts obtained from Zeng et al. [33].

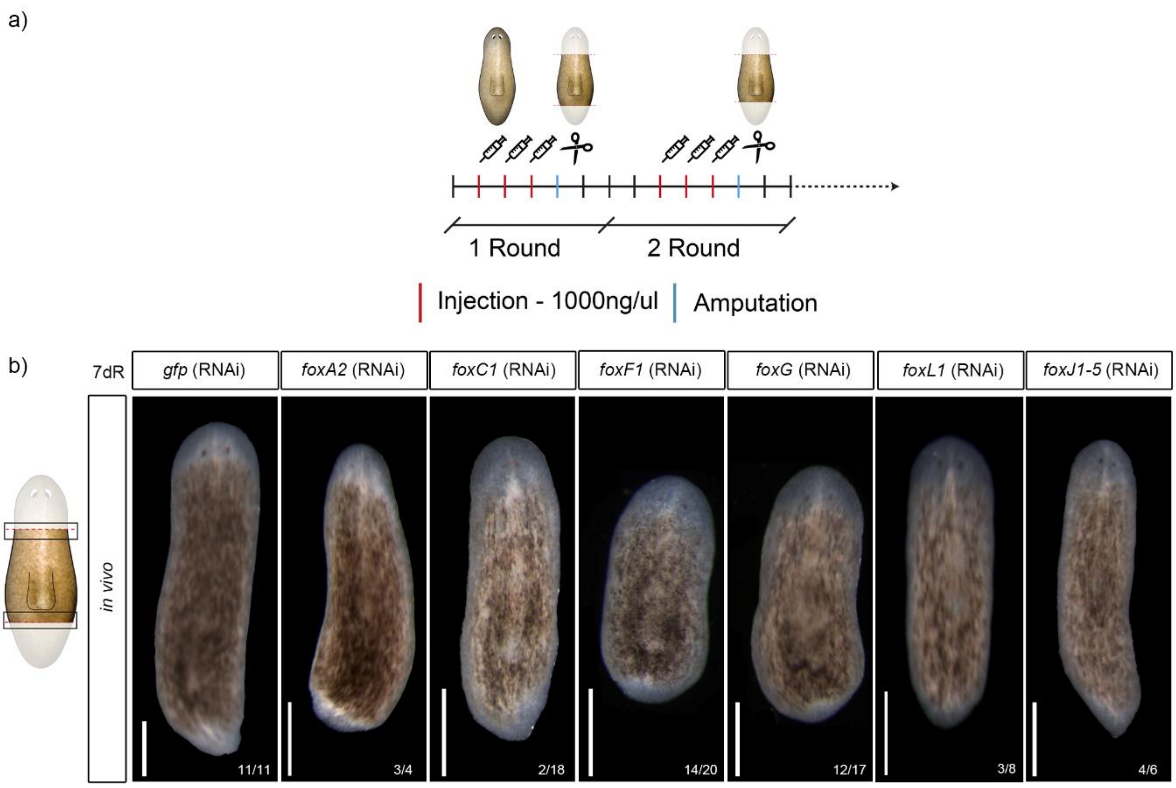

Figure S11. *fox* (RNAi) animals that presented a mild phenotype. a Schematic illustration of the RNAi procedure. **b** *in vivo* images of 7 days regenerating RNAi planarians. All of them regenerated a head, although the differentiation of the eyes was affected with a variable penetrance according to each *fox* (RNAi). *foxG* (RNAi) animals showed regenerative defects in posterior regeneration. Scale bars: 500 µm.

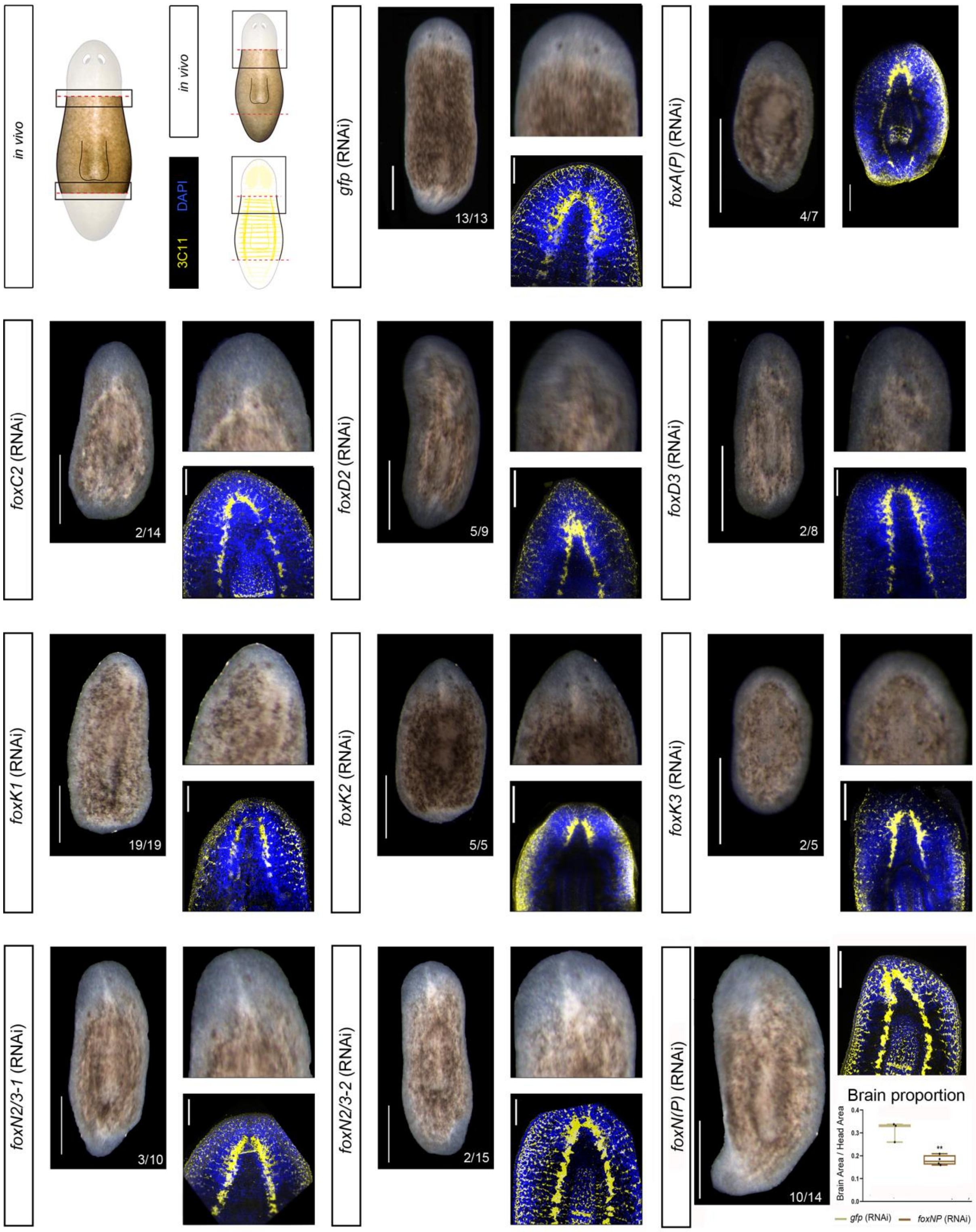

Figure S12. *fox* (RNAi) animals which presented anterior regenerative defects. For each gene is shown: an *in vivo* image at 7 days of regeneration, a magnification of the head and an immunostained head showing neural defects (anti-synapsin, 3C11). Nuclei are stained with DAPI. *foxA(P)* (RNAi) animals could not regenerate a proper blastema and after two weeks of inhibition appeared smaller than controls and with general regenerative defects. *foxNP* (RNAi) animals do not regenerate eyes and presented significative smaller

brains compared to controls (controls, n=3; RNAi, n=4; $**P<0.01$) Scale bars: 500 µm in *in vivo* images and 100 µm in confocal images.

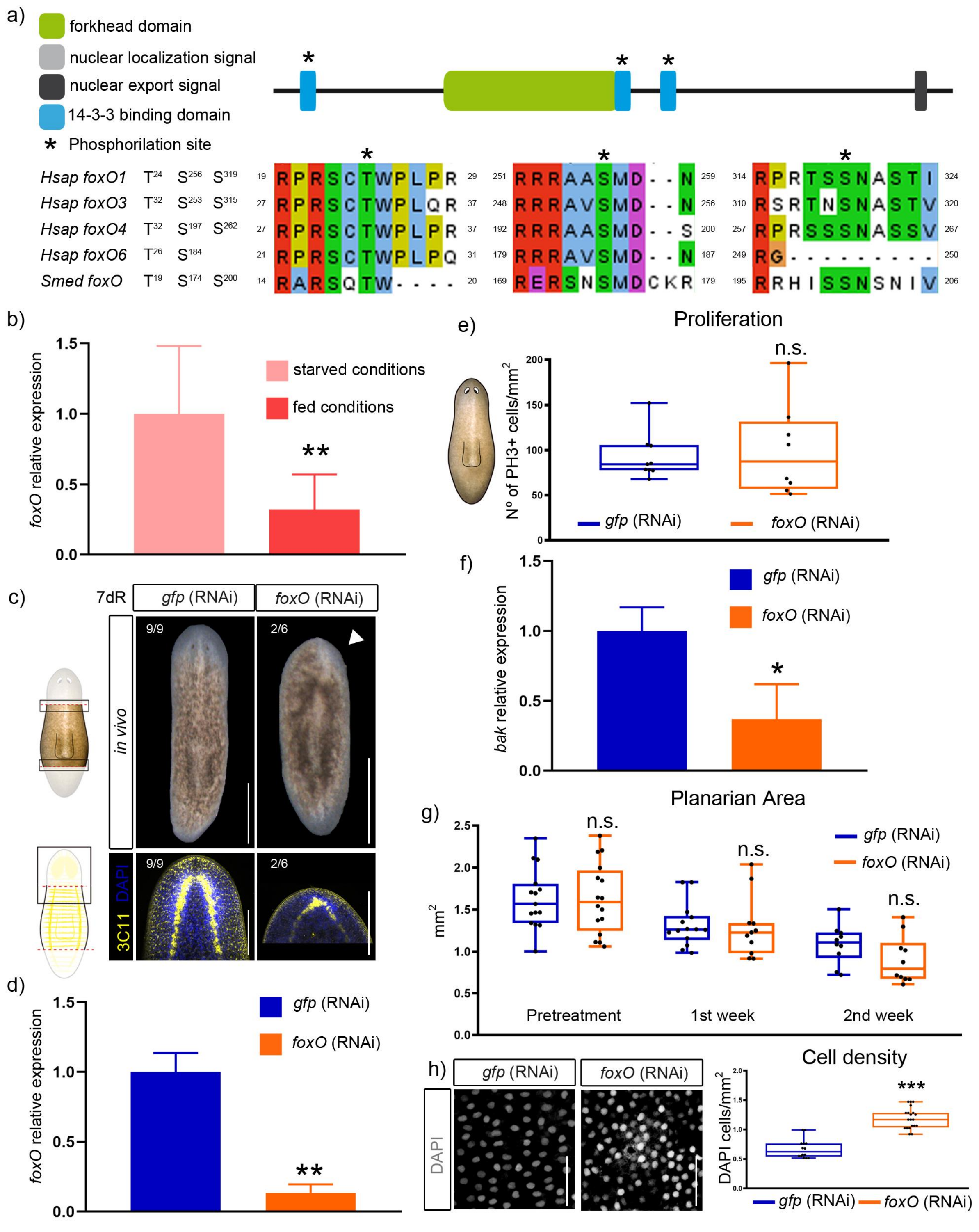

Figure S13. *Smed-foxO* inhibition does not affect cell proliferation. a Schematic illustration of conserved domains in Smed-FoxO. Alignment of FOXO amino acid sequences from *Homo sapiens* (*Hsap*) and *Schmidtea mediterranea* (*Smed*) showing high level of conservation of the three phosphorylation sites (*). **b** qRT-PCR analysis quantifying *foxO* expression in planarians that underwent starved or fed conditions, proving that *foxO* was

down-regulated in fed conditions. Relative expression is plotted as $2^{-φφφCT}$ values. Data are plotted as mean and error bars are s. d. (***P*<0.01). **c** 30% of *in vivo* animals presented regenerative defects and neural tissue (3C11) malformation. Nuclei are stained with DAPI. **d** qRT-PCR analysis quantifying *Smed-foxO* expression after one week of *Smed-foxO* inhibition, proving that it was down-regulated. Relative expression is plotted as $2^{-φφφCT}$ values. Data are plotted as mean and error bars are s. d. (***P*<0.01). **e** Quantification of PH3+ cells shows no differences between control and *Smed-foxO* (RNAi) animals after two weeks of inhibition (controls, n>7; RNAi, n>7; n.s.). In the schematic drawing the square indicates the region analysed. **f** qRT-PCR analysis quantifying *bak* expression after two weeks of *Smed-foxO* inhibition, proving that it was down-regulated. Relative expression is plotted as $2^{-φφφCT}$ values. Data are plotted as mean and error bars are s. d. (**P*<0.05). **g** Quantification of the planarian area during the RNAi treatment demonstrated no changes. **h** DAPI staining of epithelial cells in the parapharyngeal region. Quantification demonstrated an increment of the cell density (controls, n=14; RNAi, n=20; ****P*<0.001). Scale bars: c = 500 µm in *in vivo* images and 250 µm in confocal images; and h = 10 µm.

References

1. Irimia, M. *et al.* Extensive conservation of ancient microsynteny across metazoans due to cis-regulatory constraints. *Genome Res.* **22**, 2356ï 2367 (2012).
2. Rozanski, A. *et al.* PlanMine 3.0-improvements to a mineable resource of flatworm biology and biodiversity. *Nucleic Acids Res.* **47**, (2019).
3. Larroux, C. *et al.* Genesis and expansion of metazoan transcription factor gene classes. *Mol. Biol. Evol.* **25**, 980ï 996 (2008).
4. Marlétaz, F. *et al.* Amphioxus functional genomics and the origins of vertebrate gene regulation. *Nature* **564**, 64ï 70 (2018).
5. Egger, B. *et al.* A transcriptomic-phylogenomic analysis of the evolutionary relationships of flatworms. *Curr. Biol.* **25**, 1347ï 1353 (2015).
6. Wang, J. *et al.* The oyster genome reveals stress adaptation and complexity of shell formation. *Nature* **490**, 49ï 54 (2012).
7. Simakov, O. *et al.* Insights into bilaterian evolution from three spiralian genomes. *Nature* vol. 493 526ï 531 (2013).
8. Clark, A. G. *et al.* Evolution of genes and genomes on the Drosophila phylogeny. *Nature* **450**, 203ï 218 (2007).
9. Church, D. M. *et al.* Modernizing Reference Genome Assemblies. *PLoS Biol.* **9**, e1001091 (2011).
10. Mikhailov, K. V *et al.* The Genome of Intoshia linei Affirms Orthonectids as Highly Simplified Spiralians. *Curr. Biol.* **26**, 1768ï 1774 (2016).
11. Luo, Y. J. *et al.* The Lingula genome provides insights into brachiopod evolution and the origin of phosphate biomineralization. *Nat. Commun.* **6**, 8301 (2015).

12. Putnam, N. H. *et al.* Sea anemone genome reveals ancestral eumetazoan gene repertoire and genomic organization. *Science (80-.).* **317**, 86ï 94 (2007).

13. Zarrella, I. *et al.* The survey and reference assisted assembly of the Octopus vulgaris genome. *Sci. Data* **6**, 13 (2019).

14. Simakov, O. *et al.* Hemichordate genomes and deuterostome origins. *Nature* **527**, 459ï 465 (2015).

15. Adell, T. & Müller, W. E. G. Isolation and characterization of five Fox (Forkhead) genes from the sponge Suberites domuncula. *Gene* **334**, 35ï 46 (2004).

16. Grohme, M. A. *et al.* The genome of Schmidtea mediterranea highlights the plasticity of cellular core mechanisms. *Nat. Publ. Gr.* 1ï 24 (2018) doi:10.1038/nature25473.

17. Sodergren, E. *et al.* The genome of the sea urchin Strongylocentrotus purpuratus. *Science (80-.).* **314**, 941ï 952 (2006).

18. Richards, S. *et al.* The genome of the model beetle and pest Tribolium castaneum. *Nature* **452**, 949ï 955 (2008).

19. Hellsten, U. *et al.* The genome of the western clawed frog xenopus tropicalis. *Science (80-.).* **328**, 633ï 636 (2010).

20. Plass, M. *et al.* Cell type atlas and lineage tree of a whole complex animal by single-cell transcriptomics. *Science (80-.).* **1723**, eaaq1723 (2018).

21. Adler, C. E., Seidel, C. W., McKinney, S. a & Sánchez Alvarado, A. Selective amputation of the pharynx identifies a FoxA-dependent regeneration program in planaria. *Elife* **3**, e02238 (2014).

22. Vogg, M. C. *et al.* Stem cell-dependent formation of a functional anterior regeneration pole in planarians requires Zic and Forkhead transcription factors. *Dev. Biol.* **390**, 136ï 48 (2014).

23. Scimone, M. L., Lapan, S. W. & Reddien, P. W. A forkhead transcription factor is wound-induced at the planarian midline and required for anterior pole regeneration. *PLoS Genet.* **10**, e1003999 (2014).

24. Vásquez-Doorman, C. & Petersen, C. P. zic-1 Expression in Planarian Neoblasts after Injury Controls Anterior Pole Regeneration. *PLoS Genet.* **10**, (2014).

25. Scimone, M. L., Kravarik, K. M., Lapan, S. W. & Reddien, P. W. Neoblast Specialization in Regeneration of the Planarian Schmidtea mediterranea. *Stem Cell Reports* **3**, 339ï 352 (2014).

26. Scimone, M. L. *et al.* foxF-1 Controls Specification of Non-body Wall Muscle and Phagocytic Cells in Planarians. *Curr. Biol.* **28**, 3787-3801.e6 (2018).

27. He, X. *et al.* FOX and ETS family transcription factors regulate the pigment cell lineage in planarians. *Development* (2017) doi:10.1242/dev.156349.

28. Ong, T.-H. *et al.* Mass Spectrometry Imaging and Identification of Peptides Associated with Cephalic Ganglia Regeneration in Schmidtea mediterranea. *J. Biol. Chem.* jbc.M115.709196 (2016) doi:10.1074/jbc.M115.709196.

29. Lapan, S. W. & Reddien, P. W. Transcriptome analysis of the planarian eye identifies ovo as a specific regulator of eye regeneration. *Cell Rep.* **2**, 294ï 307 (2012).

30. Vij, S. *et al.* Evolutionarily Ancient Association of the FoxJ1 Transcription Factor with the Motile Ciliogenic Program. *PLoS Genet.* **8**, (2012).

31. Van Wolfswinkel, J. C., Wagner, D. E. & Reddien, P. W. Single-cell analysis reveals functionally distinct classes within the planarian stem cell compartment. *Cell Stem Cell* **15**, 326ï 339 (2014).

32. Wang, C. *et al.* Forkhead containing transcription factor Albino controls tetrapyrrole-based body pigmentation in planarian. *Cell Discov.* **2**, 16029 (2016).

33. Zeng, A. *et al.* Prospectively Isolated Tetraspanin + Neoblasts Are Adult Pluripotent Stem Cells Underlying Planaria Regeneration. *Cell* **173**, 1593-1608.e20 (2018).
